# Effects of bismuth on the microstructure and crack propagation in Sn–Ag–Cu-Bi solder joints during thermal cycling

**DOI:** 10.1007/s10854-025-15979-2

**Published:** 2025-10-24

**Authors:** C. L. Hsieh, R. J. Coyle, S. A. Belyakov, J. W. Xian, C. M. Gourlay

**Affiliations:** 1 Department of Materials, Imperial College London, London, SW7 2AZ UK; 2 Nokia Bell Labs, Murray Hill, NJ USA

## Abstract

Bismuth is added to Sn–Ag–Cu-based solders to increase strength and solder joint reliability but accelerated thermal cycling (ATC) tests have shown variable thermal fatigue performance. To better understand their thermal fatigue mechanisms, we investigate three Sn–Ag–Cu-Bi solder alloys containing between 3 and 6 wt.% bismuth in two types of ball grid array (BGA) packages tested using a harsh ATC profile of -55/125 °C. We study how the bismuth additions affect the microstructure after soldering, after ~ 4 years storage at room temperature and after thermal cycling. The Weibull reliability statistics are then correlated with the microstructural evolution including solutionising, homogenisation, bismuth precipitation, recrystallisation and crack paths. The results show that high strength Sn–Ag–Cu-Bi solders can improve the resistance to thermal fatigue, but high solder strength needs to be balanced against excessive stress transfer to the intermetallic attachment layer.

**Supplementary Information:**

The online version contains supplementary material available at 10.1007/s10854-025-15979-2.

## Introduction

There is an ongoing need to develop improved Pb-free solder alloys for evolving electronic package designs and emerging applications in harsh use environments. For high reliability end users, particularly in the automotive, aerospace and defence sectors, high thermal fatigue resistance of solder joints is a key requirement. These applications typically have longer operational life requirements, a harsher operation environment, and higher consequences of failure than consumer electronics. Therefore, we need both improved solder alloys and a deeper understanding of their stability and degradation mechanisms to build confidence in their use.

Third generation Pb-free solder alloys have been developed to target high reliability applications [1–3]. These alloys are typically near-eutectic Sn–Ag–Cu (SAC) alloys with major alloying additions of 1–6 wt.% of Bi, Sb and/or In, resulting in four to six component alloys [2, 4–6]. Generally, the alloy design rationale has been to provide increased solid solution strengthening and/or precipitation strengthening compared with Sn–Ag–Cu solders [2, 7, 8]. One sub-family of these alloys are Sn–Ag–Cu-Bi alloys, i.e. with bismuth as the only addition. It is well established that bismuth additions of 1–6 wt.% increase the creep strength of tin [9, 10] and solders such as Sn-Cu [11], Sn-Ag [12] and Sn–Ag–Cu [13–17] alloys. This occurs by solid solution strengthening [9, 17, 18] and, with sufficient Bi concentration, precipitation strengthening via the (Bi) phase [18]. However, there is much less understanding of how Sn–Ag–Cu-Bi alloys perform in ball grid array (BGA) electronic packages undergoing thermal cycling, particularly in terms of microstructure evolution and failure mechanisms.

**Fig. 1:**
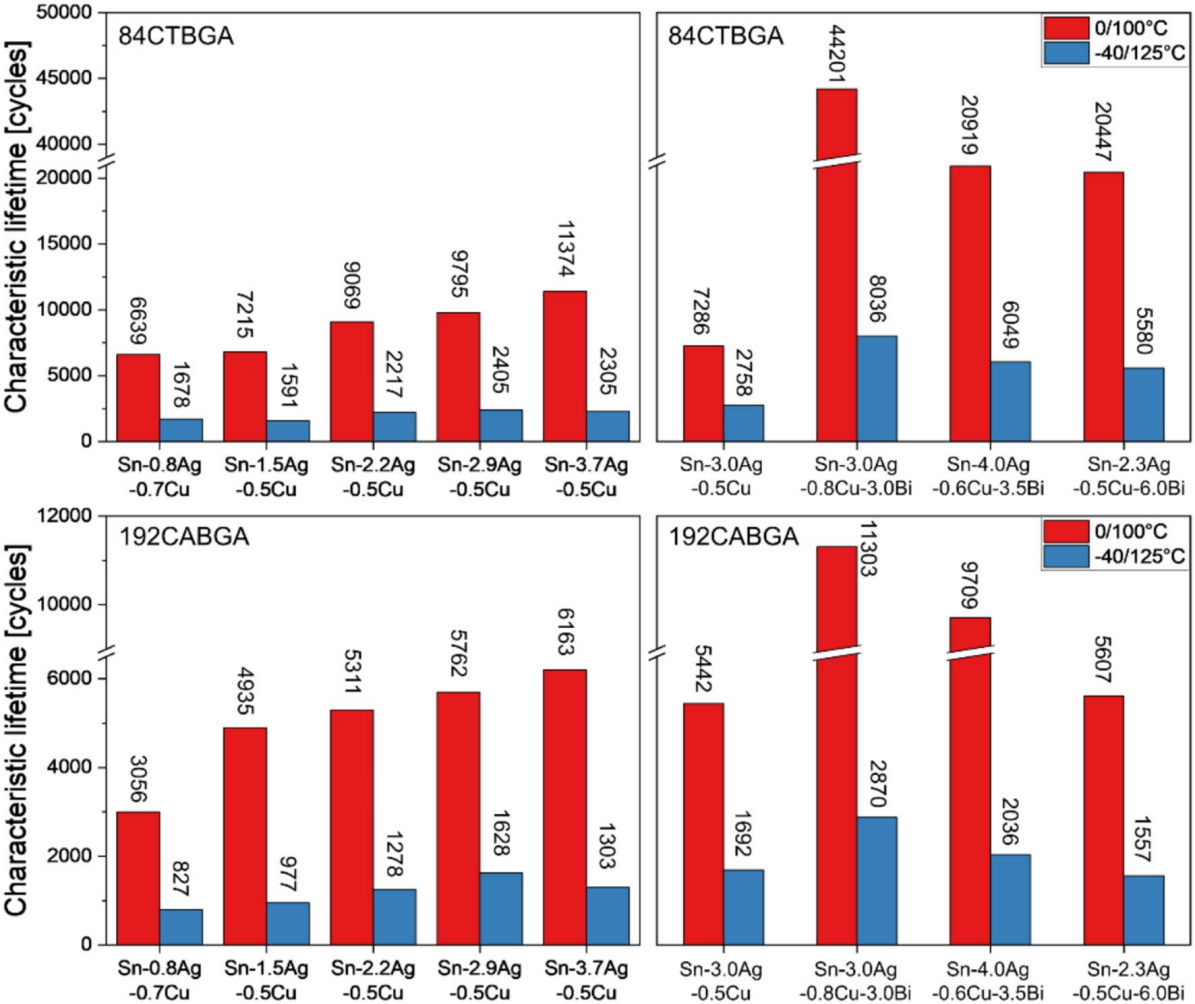
Summary of the characteristic lifetime (N63) of Sn–Ag–Cu and Sn–Ag–Cu-Bi solders in 0/100°C and 40/125°C ATC tests on 84CTBGA and 192CABGA packages. Left: Sn–Ag–Cu solder joints ordered by increasing Ag content from Ref. [24]. Right: Sn-3.0Ag-0.5Cu and Sn–Ag–Cu-Bi solder joints ordered by increasing Bi content from refs. [23] and [29]. The characteristic lifetime value of each solder alloy is labelled on top of the bar. Data on the right are from the same test vehicle as the current study. Data on the left are from a similar vehicle but with a different PCB laminate material

The thermal fatigue performance of third generation Pb-free solders has been assessed in various projects, which have shown that certain alloy compositions can significantly improve thermal fatigue resistance compared with Sn–Ag–Cu (SAC) solders [19–22]. One industrial project has run within the International Electronics Manufacturing Initiative (iNEMI) [2, 23], using daisy-chained BGA components soldered to custom test boards with more than 10 third generation Pb-free solder alloys, including accelerated thermal cycling (ATC) at various levels of harshness. The test vehicle involves an 84-pin thin core BGA (84CTBGA) and a 192-pin chip array BGA (192CABGA) package that have been used in various past ATC studies on the influence of solder alloy composition on reliability [2, 6, 24]. For example, using past data from ref. [24], Fig. 1(left) overviews the influence of Ag content on thermal fatigue resistance in Sn–Ag–Cu alloys, using the characteristic lifetime (63.2% failure (N63) from a two-parameter Weibull analysis) for Sn–Ag–Cu (SAC) alloys ranging from SAC0307 to SAC405, accounting for the final solder composition in cases where the paste and ball compositions differed. In both the 0/100 °C and -40/125 °C profiles and both package types, the characteristic life generally increases with increasing Ag content in this Ag range, similar to other work [25–27] using other package types and different thermal cycling profiles. The improved performance has been explained in terms of the increased volume fraction of eutectic Ag_3_Sn particles which increase strength [28].

The Sn–Ag–Cu-Bi solders in this iNEMI project have shown variable thermal fatigue performance compared with Sn-3.0Ag-0.5Cu. Fig. 1(right) shows the reliability data for Sn-3.0Ag-0.5Cu compared with Sn-3.0Ag-0.8Cu-3.0Bi, Sn-4.0Ag-0.6Cu-3.5Bi and Sn-2.3Ag-0.5Cu-6.0Bi. These bismuth-containing alloys performed substantially better than Sn-3.0Ag-0.5Cu in the 84CTBGA package in 0/100 °C and -40/125 °C cycling. Comparing the six-fold increase in characteristic lifetime with Bi content from 0 wt.% to 3 wt.% (Fig. 1(top right)) with the two-fold increase in characteristic lifetime with Ag content from 0.8 wt.% to 3.7 wt.% (Fig. 1(top left)), it is apparent that the different Ag contents in the three SAC + Bi solders had a limited effect on the reliability compared with their Bi additions. In the 192CABGA package, however, the SAC + Bi solders outperformed Sn-3.0Ag-0.5Cu by a smaller margin, and Sn2.3Ag-0.5Cu-6.0Bi (1557 cycles) performed slightly worse than Sn-3.0Ag-0.5Cu (1692 cycles) in -40/125 °C cycling. These results show that the relative performance of SAC + Bi solders depends on the package design and the thermal profile.

The results in Fig. 1 show the potential benefits of using Sn–Ag–Cu-Bi solders in high reliability applications. However, these solders also come with added complexity and there is a need to build a deeper understanding of how Bi additions affect the solder microstructure, damage accumulation and failure mechanisms during thermal cycling.

To explore these questions, this paper investigates the microstructures of joints in the 84CTBGA and 192CABGA packages soldered with Sn-3.0Ag-0.5Cu and the three Sn–Ag–Cu-Bi alloys in Fig. 1 in three conditions: (i) shortly after reflow soldering; (ii) after more than 4 years storage at room temperature; and (iii) after 4876 thermal cycles from -55/125 °C. This harsh thermal profile is relevant to high reliability applications in aerospace and defence. The test boards studied here were taken directly from the study reported in [23, 29, 30].

## Methods

Four alloys were investigated here, Sn-3.0Ag-0.5Cu, Sn-3.0Ag-0.8Cu-3.0Bi, Sn-4.0Ag-0.6Cu-3.5Bi, and Sn-2.3Ag-0.5Cu-6.0Bi (wt.%). The thermal cycling test vehicle included both 192CABGA and 84CTBGA packages daisy-chained to a matching daisy-chained printed circuit board (PCB). The attributes of both BGA packages and PCB used in this study are demonstrated in SI-Fig. 1 and SI-Table 1 in Supplementary Information. Further details including the stencil printing process, the reflow soldering conditions, and the thermal cycling test design can be found in References [2, 29]. The key features relevant to this paper are that the 192CABGA was soldered with 460 μm balls, the 84CTBGA with 300 mm balls, and both packages used balls and paste of the same composition. The pad finish on the packages was electrolytic Ni/Au and the surface finish on the PCB was Cu-OSP.

The electronic packages were examined in three conditions. One 84CTBGA package of each solder was examined more than 4 years after soldering after storage at room temperature. Another one 84CTBGA package and one 192CABGA package of each solder were examined after thermal cycling. To study the microstructure shortly after soldering, one 84CTBGA package of each solder was given a second reflow in a LFR400HTX TORNADO reflow oven (Surface Mount Technology, Isle of Wight, UK). The thermal profile involved heating at ~ 2 K/s to a peak temperature of ~ 240 °C and cooling at ~ 2.4 K/s to room temperature. These packages were then examined 20 h after reflow.

Thermal cycling was conducted on sample sizes of thirty-two 192CABGA and thirty-two 84CTBGA packages in accordance with the IPC-9701A guidelines [31] using a harsh -55/125 °C thermal profile with 10 K/min ramp rates and 10 min dwells at the hot and cold temperature extremes. The solder joints were monitored in situ using an event detector set at a resistance limit of 1000 ohms. All thermally cycled packages used for microstructure characterisation had experienced 4876 thermal cycles from -55/125 °C.

BGA packages were cut from the test vehicles, mounted in Struers VersoCit II cold mounting resin, wet ground to 4000 grit SiC paper to near the centre of a row of joints, and then polished with colloidal silica. Analytical scanning electron microscopy (SEM) was conducted on a Zeiss SIGMA field-emission gun SEM (Carl Zeiss, Oberkochen, Germany) with an Oxford Instruments INCA x-sight energy dispersive x-ray (EDX) detector (Oxford Instruments, Oxfordshire, UK) and a Bruker electron backscatter diffraction (EBSD) detector (Bruker AXS Inc., Fitchburg, WI, USA). Cross-sections were carbon coated before EBSD acquisition. EBSD maps were analysed within Bruker Esprit 2.1 and with the MTEX toolbox in MATLAB.

Some soldered packages were investigated by differential scanning calorimetry (DSC) to study the effects of Bi concentration on the nucleation undercooling for β-Sn during solidification. Samples were prepared by slicing packages into a region of 3 × 2 joints and then grinding the PCB side to ~ 100 μm thickness. This was then placed PCB side down in an Al pan in a heat flux DSC instrument (Mettler Toledo DSC1) under a nitrogen atmosphere. The liquidus temperature of joints was measured by the cyclic DSC method described in references [32–34]. Nucleation temperatures were measured by heating and cooling a package at 20 K/min and taking the onset on cooling as the β-Sn nucleation temperature. The nucleation undercooling for β-Sn was defined as the difference between the β-Sn liquidus temperature measured by cyclic DSC experiments, and the onset on cooling. Since DSC was performed on soldered packages, the undercooling measurements account for the change in liquid composition due to the dissolution of the Cu and Ni/Au substrates, as well as the role of the intermetallic reaction layers on the nucleation of β-Sn [35–40].

## Results and discussion

### Microstructures before thermal cycling

**Fig. 2:**
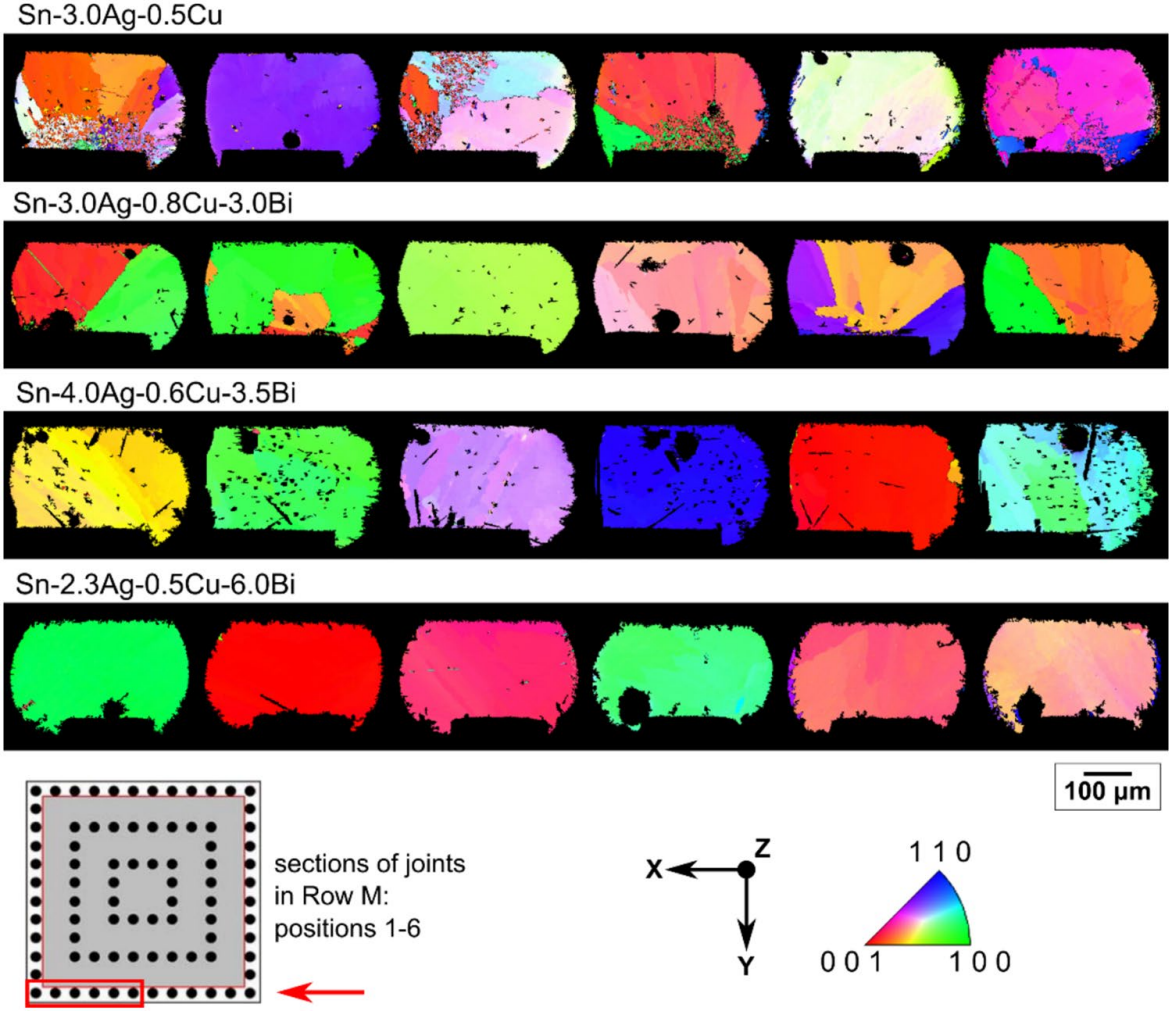
EBSD orientation maps (β-Sn IPF-X) of the M1–M6 joint cross-sections from 84CTBGA packages after soldering

Fig. 2 shows the microstructures of the joints at locations M01–M06 for each solder alloy after reflow soldering (at time zero) in the 84CTBGA package. The joints had one of three types of β-Sn microstructure: single grain joints, multi-grain joints with some interlacing, and multi-grain joints without interlacing in the cross-section. This is consistent with past work on Sn–Ag–Cu solders [41–45]. However, the Bi concentration in the alloy significantly affected the proportion of joints with each type of microstructure. Fig. 2 shows that Bi additions promoted single grain joints and suppressed interlacing.

**Fig. 3:**
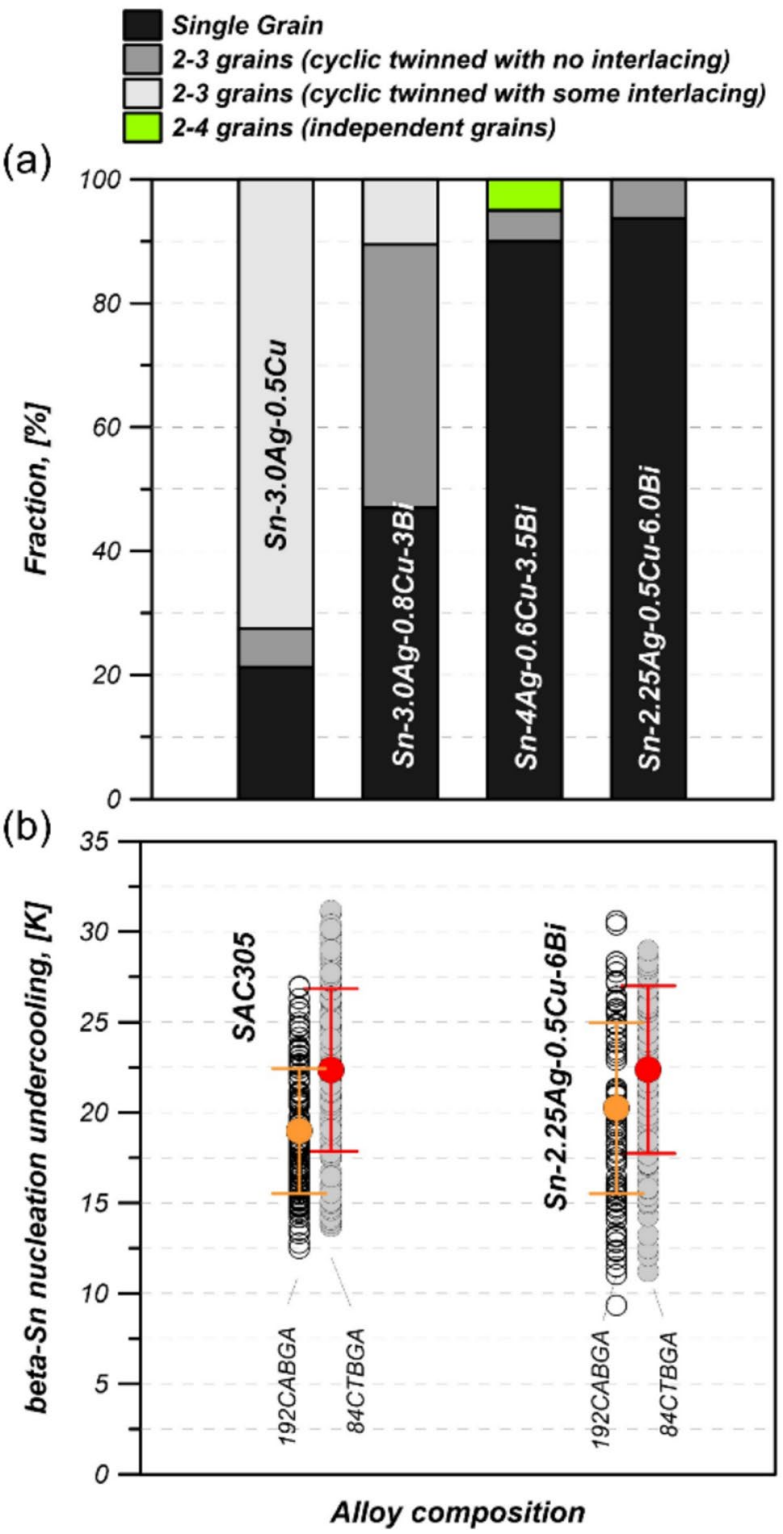
**a** Summary of the β-Sn microstructure after soldering in the four alloys based on EBSD analysis of 84CTBGAs. At least 12 joints were studied for each composition. **b** β-Sn nucleation undercooling from DSC experiments on 3 × 2 regions of joints cut from 84CTBGA and 192CABGA packages. Shown are datapoints for individual joints with the mean ± standard deviation overlaid

The percentage of joints containing each type of β-Sn microstructure is summarised statistically in a stacked bar chart in Fig. 3(a) from at least 12 joints from the 84CTBGA package for each composition. For the alloys with 3.5 and 6wt% Bi, > 90% of joints contained a single β-Sn grain compared with Sn-3.0Ag-0.5Cu where only ~ 20% of joints were single grain. In (Bi-free) Sn–Ag–Cu joints, past work has found that multi-grain joints and interlacing are related to the nucleation undercooling for the β-Sn phase [43]. Fig. 3(b) shows nucleation undercooling data for the Sn-3.0Ag-0.5Cu and Sn-2.3Ag-0.5Cu-6.0Bi joints from DSC measurements on 3 × 2 joint regions of the packages. The undercooling values are similar for the two solders, indicating that the Bi addition did not affect the undercooling sufficiently to account for the substantial increase in the fraction of single grain joints. Thus, Bi additions do not seem to affect the β-Sn nucleation undercooling significantly when soldered to Cu and Ni/Au substrates and the promotion of single grain joints was not due to undercooling differences in this case. A further dedicated study is required to understand how and why Bi promotes single grain joints and suppresses interlacing. Fig. 3(b) also shows that, for both solders, the undercooling is slightly deeper for the 84CTBGA package than the 192CABGA package. This is likely to be related to the smaller balls (300 versus 460 μm) in the 84CTBGA package versus the 192CABGA, consistent with past work on SAC solders [38, 46, 47].

**Fig. 4:**
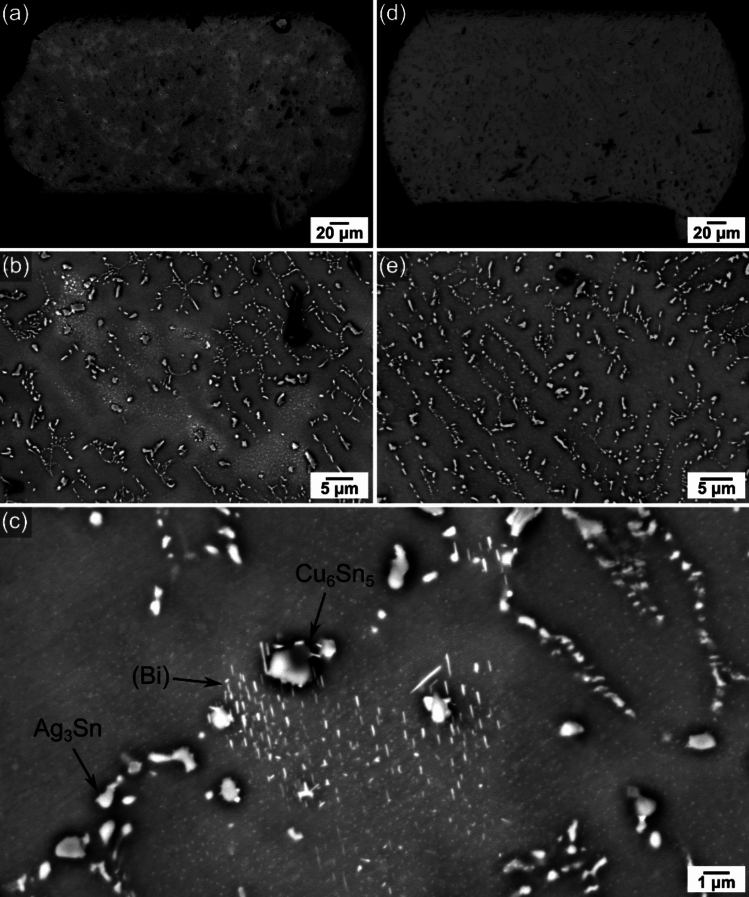
Microstructure evolution at room temperature in Sn-3.0Ag-0.8Cu-3.0Bi joints. **a**–**c** 20 h after reflow soldering. **d**–**e** after 4 years storage at room temperature

 Fig. 4(a)-(c) show the typical microstructure of a Sn-3.0Ag-0.8Cu-3.0Bi joint 20 h after reflow soldering. At low magnification in Fig. 4(a), there are variations in greyscale across the joint, and the higher magnification images in Fig. 4(b) and (c) reveal that the brighter areas are regions with (Bi) precipitates. This indicates a highly non-uniform distribution of (Bi) phase across the joint 20 h after soldering, consistent with eutectic growth and solid-state precipitation. Similar images of joints 20 h after reflow soldering in Sn-4.0Ag-0.6Cu-3.5Bi and Sn-2.3Ag-0.5Cu-6.0Bi are given in SI-Fig. 2 and 3 in the Supplementary Information and show a similar phenomenon.

After > 4 years of storage at room temperature, significant changes in the (Bi) phase had occurred. In Fig. 4(d)-(e), a more uniform grey scale is apparent across the joint at low magnification and much less (Bi) phase is present compared with 20 h after soldering in Fig. 4(a)-(c). Images of joints 4 years after soldering in Sn-4.0Ag-0.6Cu-3.5Bi and Sn-2.3Ag-0.5Cu-6.0Bi are given in SI-Fig. 2 and 3 in the Supplementary Information. They also have a more uniform grey scale across the joint, the Sn-4.0Ag-0.6Cu-3.5Bi example has a very low fraction of (Bi) phase, and the Sn-2.3Ag-0.5Cu-6.0Bi has a significantly higher fraction of (Bi) phase distributed across the whole joint.

Cross-sections from four to twelve joints were examined for each solder alloy to check the reproducibility of the findings. All cross-sections from all three Bi-containing solders contained (Bi) phase 20 h after soldering. After 4 years at room temperature, in Sn-3.0Ag-0.8Cu-3.0Bi joints only ~ 10% of cross-sections examined contained discernible (Bi) phase, in Sn-4.0Ag-0.6Cu-3.5Bi joints ~ 80% of cross-sections contained (Bi), and in Sn-2.3Ag-0.5Cu-6.0Bi joints all cross-sections contained (Bi) phase. In the 10% and 80% cases, the volume fraction of (Bi) phase was sufficiently low that (Bi) was absent in some cross-sections.

**Fig. 5:**
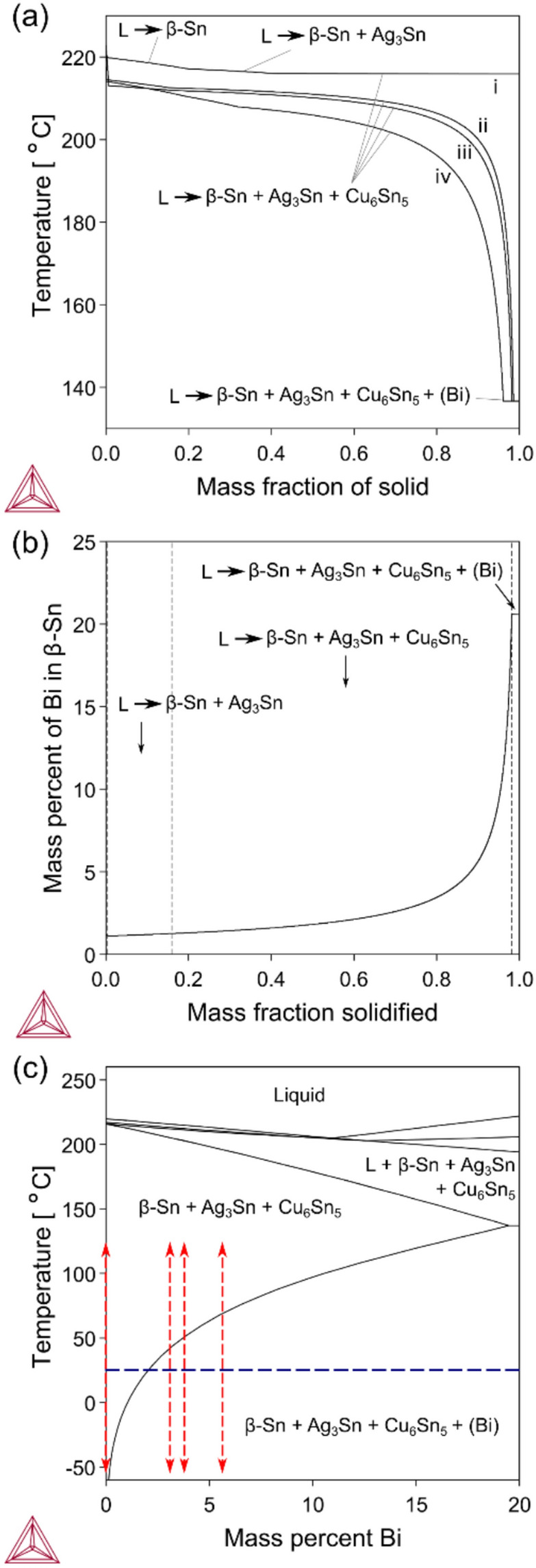
Thermodynamic calculations with the NIST solders database [48]: **a** Solidification paths assuming the Scheil model: (i) Sn-3.0Ag-0.5Cu, (ii) Sn-3.0Ag-0.8Cu-3.0Bi, (iii) Sn-4.0Ag-0.6Cu-3.5Bi, (iv) Sn-2.3Ag-0.5Cu-6.0Bi. **b** Bi concentration profile in β-Sn after Scheil solidification of Sn-4.0Ag-0.6Cu-3.5Bi. **c** (97.25-x)Sn-2.3Ag-0.5Cu-xBi isopleth. Vertical red lines mark the thermal cycling range in the four alloys. The horizontal blue line marks room temperature 25 °C

These microstructural features and changes during extended room temperature storage are next explored with thermodynamic calculations using the NIST solders database with Thermo-Calc 2019a software [48]. Fig. 5(a) is a plot of solid fraction versus temperature during solidification of the four alloys, assuming Scheil conditions. Scheil condition refers to the assumption of no solute diffusion within the solid, while solute diffusion in the liquid is considered infinitely fast [49]. From this figure, we see that the Bi additions of 3–6 wt.% (i) depress the β-Sn liquidus temperature (the intercept of the curves with y-axis at mass fraction of solid = 0), (ii) cause the L → β-Sn + Ag_3_Sn + Cu_6_Sn_5_ eutectic reaction to occur over a temperature range (the span of the curves labelled with L → β-Sn + Ag_3_Sn + Cu_6_Sn_5_ in y-direction), and (iii) result in a small fraction of a nonequilibrium eutectic containing the (Bi) phase at ~ 137 °C (the flat segments of the curves near mass fraction of solid = 1).

 Fig. 5(b) shows how the Bi content varies within β-Sn during solidification due to microsegregation assuming the Scheil model, using Sn-4.0Ag-0.6Cu-3.5Bi as an example. In this figure, mass fraction solidified = 0 represents the β-Sn solidified at the onset of solidification, while mass fraction solidified = 1 represents the β-Sn solidified at the end of solidification. The dashed lines divide the figure into three sections corresponding to three eutectic reactions during the solidification of Sn-4.0Ag-0.6Cu-3.5Bi. The Scheil model assumes (i) no solute diffusion in the solid and infinitely fast solute diffusion in the liquid, and (ii) local equilibrium at the solid–liquid interface [49]. Under these assumptions, the composition of β-Sn formed during solidification follows the solidus line in the SAC + Bi phase diagram (Fig. 5(c)). Accordingly, β-Sn contains only ~ 1 wt.% Bi at the onset of solidification (mass fraction solidified = 0 in Fig. 5(b)), while the Bi content in as-solidified β-Sn gradually increases during solidification, reaching ~ 21 wt.% Bi in the last β-Sn to solidify (mass fraction solidified = 1 in Fig. 5(b)). The large variation in Bi content will exist over the length scale of half the secondary dendrite arm spacing [50–52], leading to significant Bi concentration gradients in β-Sn. This contrasts with Ag and Cu solute which have near-zero concentration in β-Sn and shallow gradients [48].

On cooling to room temperature, all of the Bi-containing solders drop below the β-Sn solvus (Fig. 5(c)). The retrograde shape of the β-Sn solvus means that regions of β-Sn with higher Bi content will be both more supersaturated with respect to the equilibrium concentration of Bi in β-Sn at room temperature, and more undercooled with respect to the β-Sn solvus. Thus, the largest driving force for (Bi) phase precipitation is in the β-Sn that solidified last.

Using these thermodynamic calculations in Fig. 5(a)-(c), the microstructures 20 h after soldering can be understood. The presence of some eutectic (Bi) in all alloys is due to limited back diffusion of Bi solute in β-Sn during solidification, similar to the calculations of microsegregation with the Scheil model in Fig. 5(a). The highly non-uniform solid-state precipitation of (Bi) plates in the first 20 h after soldering (e.g. Fig. 4(a)) is due to the large Bi concentration gradients in β-Sn created by solidification (Fig. 5(b)) which creates areas of higher Bi content that are more supersaturated at room temperature.

After four years of storage at room temperature, the significant reduction in volume fraction of the (Bi) phase is due to diffusion causing partial homogenisation of Bi solute in β-Sn and partial solutionising. The large Bi concentration gradients (Fig. 5(b)), coupled with the high homologous temperature of T/T_m_ ~ 0.6 at room temperature drove diffusion and microstructural change. Thus, a key finding of this study is that 3–6 wt.% Bi additions to SAC solders result in an alloy that undergoes substantial microstructural change at room temperature.

### Microstructure and damage evolution in thermal cycling

**Fig. 6:**
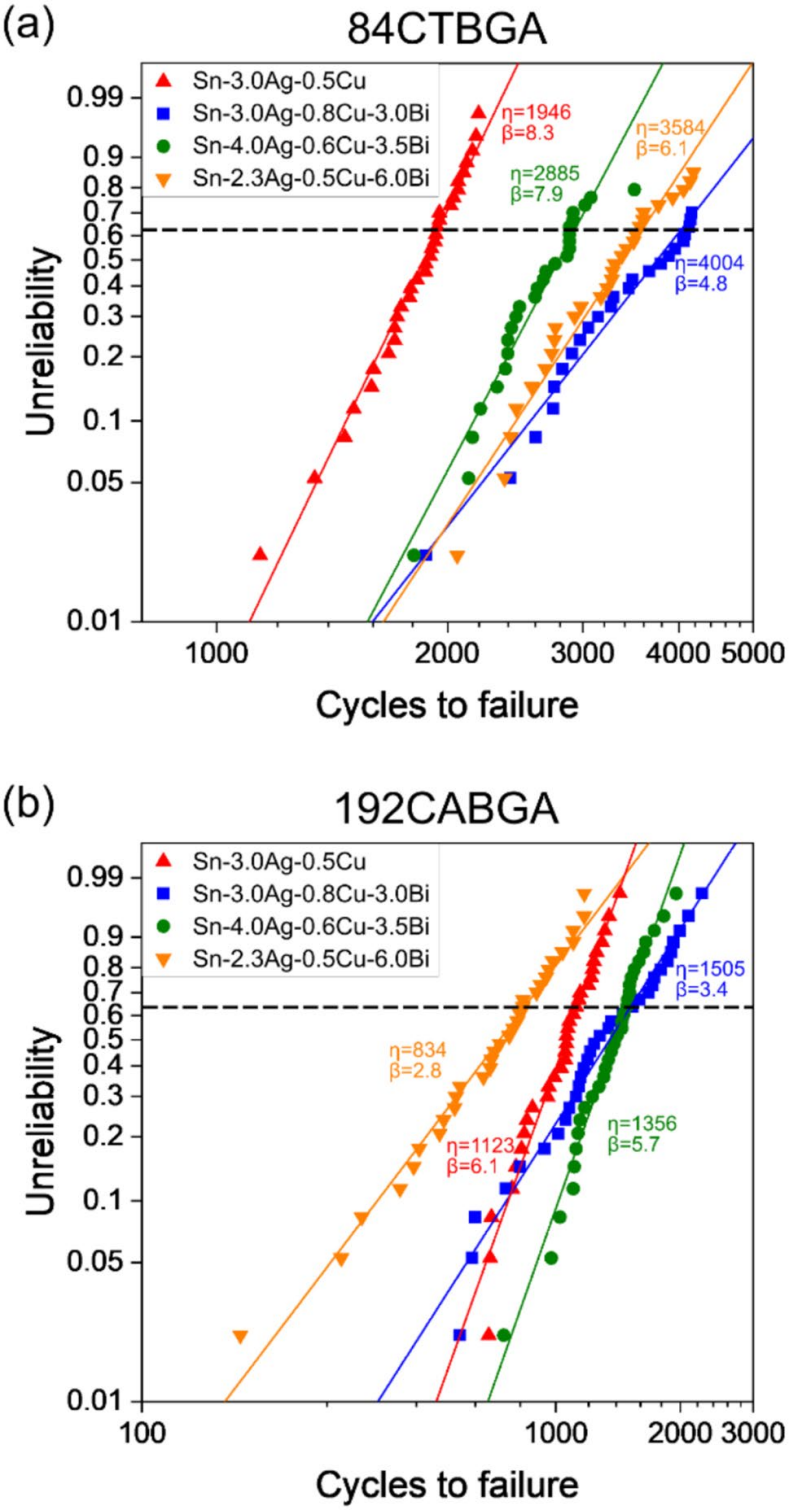
Thermal fatigue performance of Sn–Ag–Cu and Sn–Ag–Cu-Bi solders in -55/125 °C ATC tests. Weibull plots of three Sn–Ag–Cu-Bi solders and SAC305 in **a** 84CTBGA and **b** 192CABGA packages. The horizontal dashed line indicates 0.63 unreliability. The characteristic lifetime and the shape parameter of each alloy are labelled as η and β, respectively

Weibull plots for -55/125 °C thermal cycling of 84CTBGA and 192CABGA packages soldered with SAC305 and the three SAC + Bi alloys are given in Fig. 6. In the 84CTBGA packages in Fig. 6(a), the Bi-containing solders significantly outperformed SAC305 in terms of both characteristic life and 1% cumulative failure. This relative performance of the four alloys is similar to the less harsh ATC tests in Fig. 1, where, in the top right figure, the N63 lifetime of SAC + Bi solders are at least two times higher than that of SAC305. There is also a reduction in characteristic life in all alloys in Fig. 6 compared with Fig. 1. For instance, the characteristic lifetime of Sn-2.3Ag-0.5Cu-6.0Bi is 20,447 cycles for 84CTBGA package in 0/100 °C thermal cycling (Fig. 6(a)), while that is reduced to 3584 cycles in -55/125 °C thermal cycling (Fig. 1(top right)). This is due to the increased harshness of the − 55/125 °C ATC profile compared with the 0/100 and − 40/125 °C profiles. On the other hand, the Weibull plot of the 192CABGA packages in Fig. 6(b) shows that the highest Bi solder, Sn-2.3Ag-0.5Cu-6.0Bi, had a lower characteristic life and lower Weibull slope than SAC305 and, while the Sn-3.0Ag-0.8Cu-3.0Bi and Sn-4.0Ag-0.6Cu-3.5Bi solders had higher characteristic life than SAC305, their first failure and 1% cumulative failure are fairly similar to SAC305. The results in Fig. 6 are explored next by studying the microstructure and damage after 4876 cycles.

**Fig. 7:**
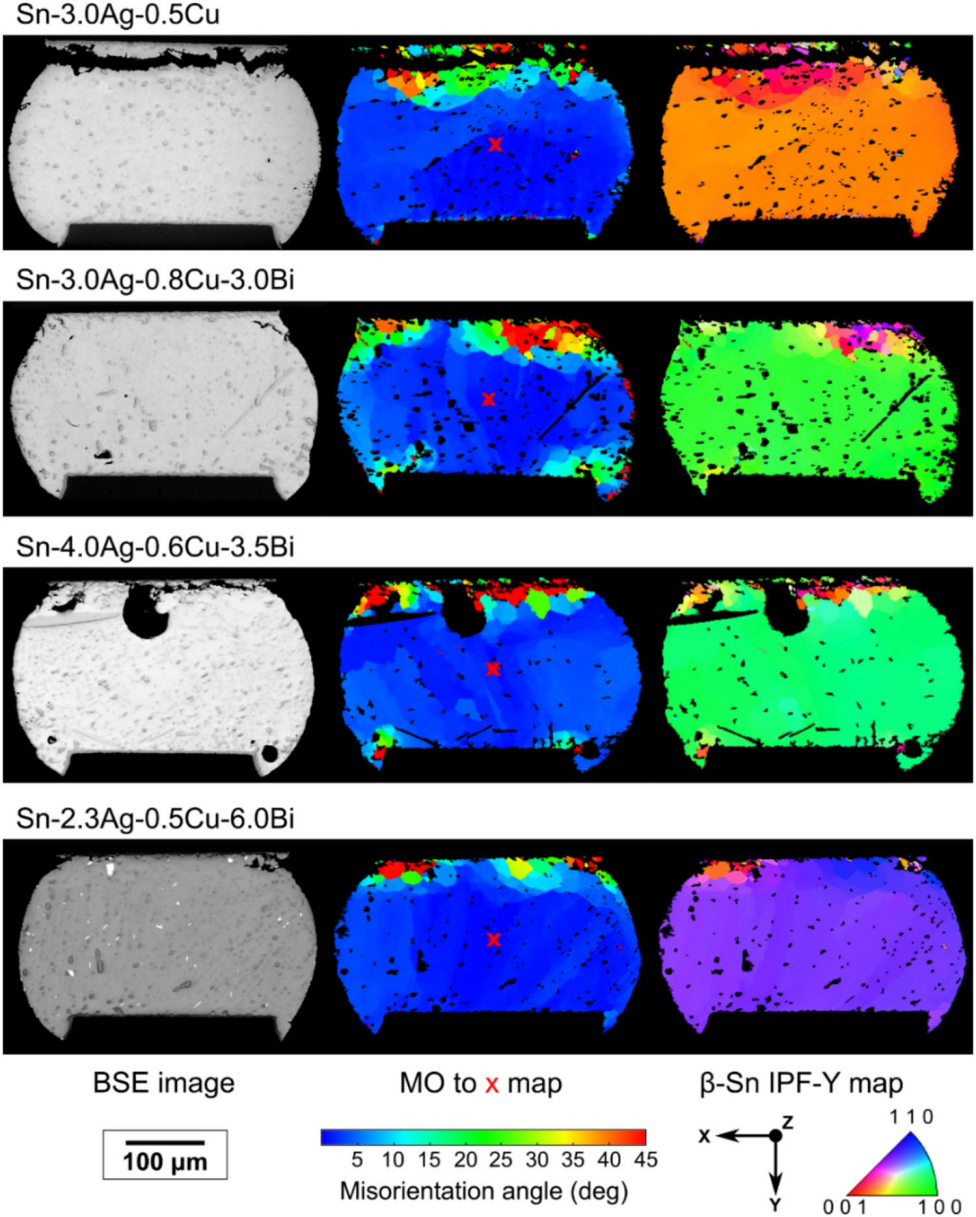
EBSD mapping of typical single grain joints from the four alloys in the 84CTBGA after 4876 cycles from -55/125 °C. First column: backscattered SEM images. Second column: maps of misorientation with respect to the orientation of the central red cross. Third column: orientation maps with IPF-Y colouring

 Fig. 7 overviews the typical deformation microstructures in 84CTBGA solder joints of Sn-3.0Ag-0.5Cu and the three SAC + Bi alloys after 4876 cycles from -55/125 °C. A single grain joint was selected as the example for each solder to simplify the comparison. The Sn-3.0Ag-0.5Cu joint is fully cracked through the β-Sn at the top (package) side with full detachment indicating that 4876 cycles involved many thermal cycles after this joint had failed electrically, consistent with the Weibull plot in Fig. 6(a). The SAC + Bi alloys are each partially cracked at the top (package) side. Despite the differences in cracking extent, the joints from each alloy contain similar deformation microstructures: the joints contain regions of localised misorientation and recrystallisation in the β-Sn at the top (package) side with fewer localised misorientations at the bottom (PCB) side. For Sn-3.0Ag-0.5Cu, this result is consistent with past studies [24, 53, 54], as well as a recent multi-scale model of Sn-3.0Ag-0.5Cu in this 84CTBGA package [55] which showed how plastic strain and stored energy localise in the β-Sn near the top (package side) interface.

Creep may also play an important role in the damage of solder joints, as solder joints are dwelled at high homologous temperature (~ 0.8 T_m_) for 10 min in each thermal cycle. Creep may occur through the sliding of recrystallized β-Sn grain boundaries, resulting in localised creep deformation at the top part of solder joints. Although grain boundary sliding is not studied in this work, it has been reported in previous studies, including sliding at recrystallized β-Sn grain boundaries [56, 57], and sliding at pre-existing β-Sn grain boundaries [58, 59]. Apart from grain boundary sliding, dislocation creep may also operate in solder joints during thermal cycling [60, 61]. These creep mechanisms may accelerate the failure of solder joints during thermal cycling. However, in the scope of this work, it is difficult to assess the contribution of creep to joint failure, and the dominant creep mechanism of SAC + Bi solders during thermal cycling is unclear. It is important for future studies to further investigate the creep mechanisms of SAC + Bi joints during thermal cycling.

**Fig. 8:**
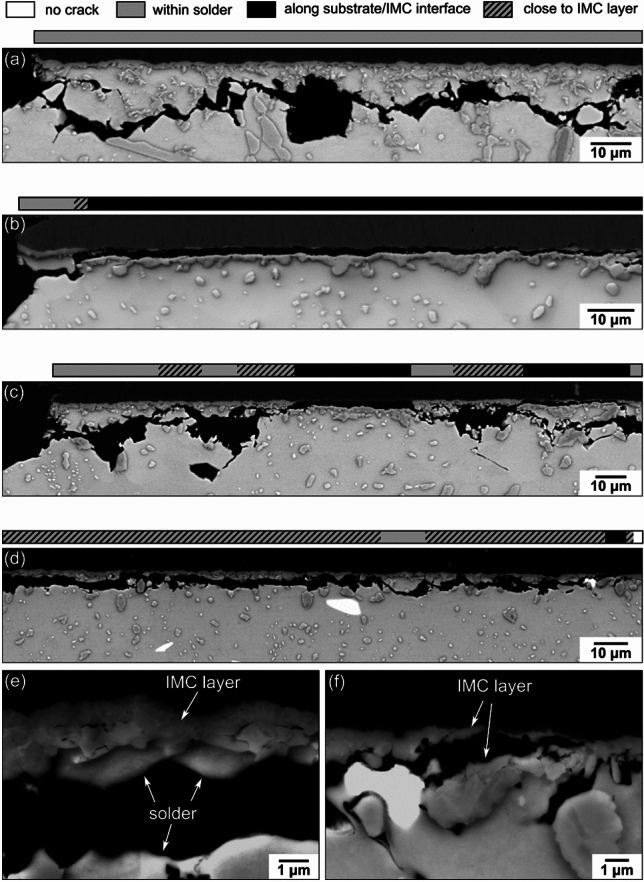
Examples of crack paths in thermally cycled Sn–Ag–Cu-Bi solder joints. **a** Crack path fully within the solder. **b** Crack path mostly along the substrate/IMC layer interface. **c** Mixed crack path both within the solder and along the substrate/IMC layer interface. **d** Crack path very close to the IMC layer through both the β-Sn (e.g. (**e**)) and parts of the IMC layer (e.g. (**f**)). The horizontal bars above **a**–**d** indicate the changing type of crack path in each case using shading

**Fig. 9:**
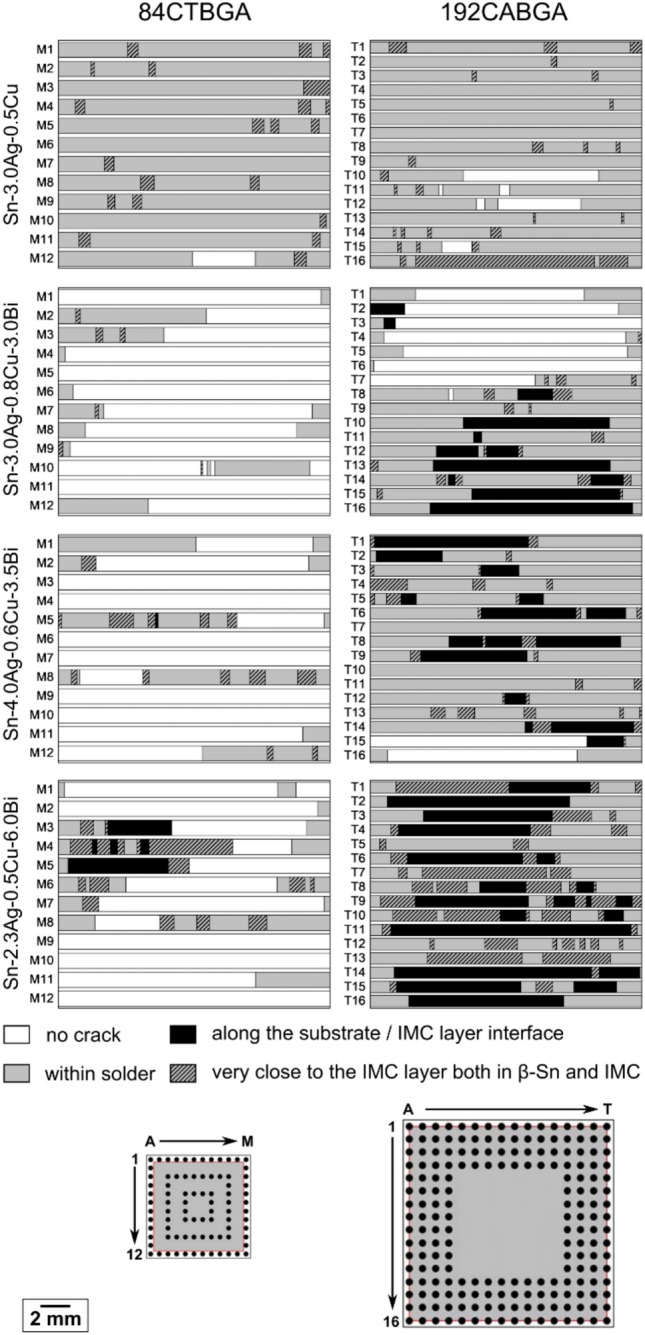
Overview of crack paths in one cross-section of each joint in the outer row of one 84CTBGA and one 192CABGA package of the four solders, based on the method shown in Fig. 8

An important difference between Sn-3.0Ag-0.5Cu and the three SAC + Bi solders was that the Bi additions caused a change in crack path in some joints. In the thermally cycled Sn–Ag–Cu-Bi solder joints, four types of cracking mode were identified: (i) crack paths completely within the solder (Fig. 8(a)); (ii) crack paths along the interface between the substrate and the IMC layer (Fig. 8(b)); (iii) crack paths very close to the IMC layer (Fig. 8(d)) involving a mixture of crack propagation through the solder (Fig. 8(e)) and some fracture through the IMC layer (Fig. 8(f)); and (iv) crack paths involving combinations of (i), (ii) and (iii) (Fig. 8(c)). To more clearly visualise the individual cracking behaviour in each cross-section, a bar is plotted with shading to indicate the different types of crack path at the top of each SEM image from Fig. 8(a) to (d). Based on this method, Fig. 9 summarizes the crack paths in one cross-section of all joints in the outer row of one 84CTBGA and one 192CABGA package of the four solders.

Comparing the crack paths from a single outer row of joints in Fig. 9 with the Weibull distributions from 32 packages in Fig. 6, the following correlations can be seen. First, in the 84CTBGA, all three SAC + Bi solders outperformed Sn-3.0Ag-0.5Cu (Fig. 6) and, generally, their crack paths alternated repeatedly either between the bulk solder and near the IMC layer (Sn-3.0Ag-0.8Cu-3.0Bi and Sn-4.0Ag-0.6Cu-3.5Bi) or between the bulk solder, near the IMC layer and in the IMC layer (Sn-2.3Ag-0.5Cu-6.0Bi) and all involved a high length fraction of crack through the solder. Second, in the 192CABGA, the Sn-3.0Ag-0.8Cu-3.0Bi and Sn-4.0Ag-0.6Cu-3.5Bi solders outperformed Sn-3.0Ag-0.5Cu but by a smaller margin than in the 84CTBGA (Fig. 6) and some of these joints had continuous cracking along the substrate/IMC layer interface, spanning 40% to 75% of the cross-sectional joint width, with solder cracking spanning > 25% of the cross-sectional joint width (Fig. 9). Third, in the 192CABGA, the Sn-2.3Ag-0.5Cu-6.0Bi had a lower characteristic life than Sn-3.0Ag-0.5Cu (Fig. 6) and its joints contained long and continuous cracks along the substrate/IMC layer interface, sometimes spanning > 85% of the cross-sectional joint width, and with solder cracking less than 10% of cross-sectional joint width (Fig. 9).

**Fig. 10:**
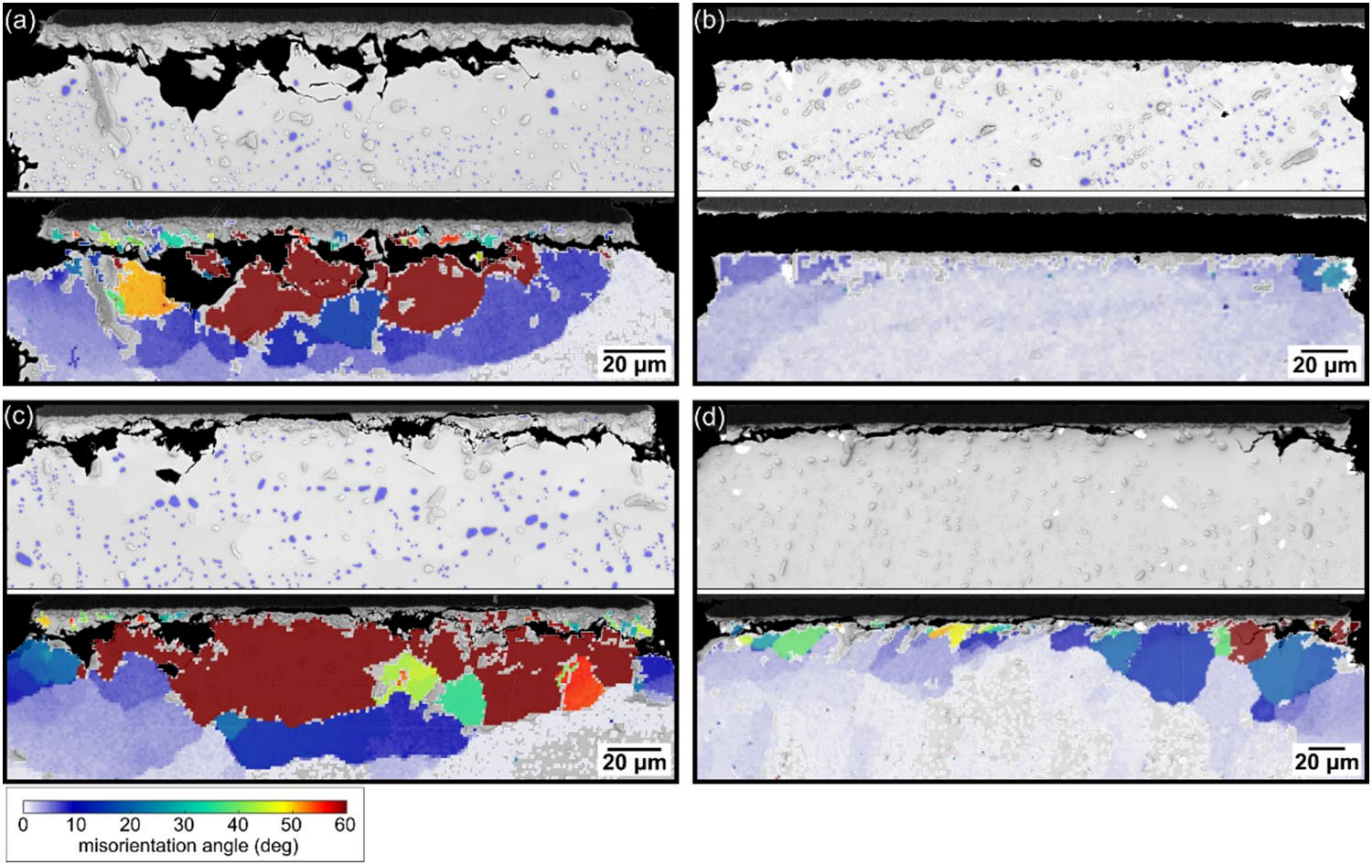
Relationships between crack paths and damage accumulation in the β-Sn phase shown using pairs of BSE images and EBSD misorientation (MO) maps. **a** Crack path mostly within the solder with an extensive area of MO and recrystallisation in β-Sn. **b** Crack path mostly along the substrate/IMC layer interface with low MO and no recrystallisation in the β-Sn. **c** Crack path alternating between the solder and the substrate/IMC layer interface with an extensive area of MO and recrystallisation in β-Sn. **d** Crack path very close to the IMC layer, with subgrains and recrystallisation in β-Sn. In **a**–**c**, Ag_3_Sn particles are masked in blue to highlight regions with accelerated coarsening. The examples are from Sn–Ag–Cu-Bi joints after 4876 cycles from -55/125 °C

To understand how relatively good solder joint life resulted from mixed-mode cracking with a repeatedly alternating crack path or sufficient solder cracking, EBSD mapping was performed on a range of Sn-3.0Ag-0.8Cu-3.0Bi and Sn-2.3Ag-0.5Cu-6.0Bi joints. Fig. 10(a) shows a crack path primarily within the solder in a Sn-3.0Ag-0.8Cu-3.0Bi 84CTBGA joint, where a region of recrystallized β-Sn grains spanned most of the top width (brown and orange grains in the EBSD misorientation map) with highly coarsened Ag_3_Sn particles in the recrystallized area, and the crack has propagated along the recrystallized grain boundaries. The large area of high misorientation and recrystallization is associated with stored energy from the cyclic plastic deformation of β-Sn that localises near the package side [41, 62, 63]. This is aided by accelerated coarsening of Ag_3_Sn in these regions which reduces strength and arises from strain-enhanced Ag_3_Sn coarsening [53, 64] and accelerated diffusion along recrystallized grain boundaries [53, 54, 65]. Since solder cracks normally propagate along the high angle grain boundaries of recrystallized grains [41, 66–68], sufficient stored energy must normally build up within β-Sn from cyclic plasticity for recrystallization to occur before the cracks can propagate in the solder.

In contrast, Fig. 10(b) shows a long and continuous crack path along the substrate/IMC layer interface in a Sn-2.3Ag-0.5Cu-6.0Bi 192CABGA joint. We see that the Ag_3_Sn particles in the β-Sn remain much smaller and more uniformly distributed, the β-Sn is significantly less misoriented from the parent grain and there is no recrystallization below the crack path in this example. That is to say, there is limited plastic deformation of β-Sn and significantly less energy has been absorbed by the β-Sn phase. The crack path along the substrate/IMC layer is also relatively straight in contrast to solder fatigue cracks which propagate with a tortuous path along recrystallized grain boundaries.

Fig. 10(c) shows a Sn-3.0Ag-0.8Cu-3.0Bi 84CTBGA joint with the crack path alternating between the solder and the substrate/IMC layer interface. The β-Sn is highly recrystallized with misorientations from light green (MO ~ 30°) to deep red/brown (MO > 60°) in the EBSD misorientation maps, and the Ag_3_Sn particles are significantly coarsened in this region. This indicates that this type of mixed crack path involves cyclic plastic strain accumulation in the β-Sn, followed by recrystallisation before the cracks propagated, similar to solder cracks.

Fig. 10(d) shows the M4 joint of Sn-2.3Ag-0.5Cu-6.0Bi in the 84CTBGA, with a continuous crack path very close to the IMC layer on the left side of the cross-section. Below this crack path, there is some β-Sn recrystallization with misorientations up to 50° (yellow grain). This shows that, even though this type of crack path is very close to the IMC layer and involves fracture through both the solder (e.g. Fig. 8(e)) and the IMC layer (e.g. Fig. 8(f)), there was sufficient plastic deformation in the β-Sn to drive recrystallization of the β-Sn.

Two factors that determine the degree of cracking along the substrate/IMC layer interface during thermal cycling are (i) the yield/flow stress of the solder and (ii) the maximum thermal strain per cycle. Using a solder with higher yield/flow stress reduces the plastic deformation and energy dissipation in the β-Sn phase, and transfers a higher stress to the IMC layer, increasing the likelihood of cracking along the substrate/IMC layer interface. Increasing the Bi concentration in SAC + Bi solders increases the yield stress [69, 70] and thus, higher Bi concentrations would be expected to transfer more stress to the IMC layer. Similarly, a higher maximum thermal strain per cycle increases the stress transferred to the IMC layer. The large ∆T of the harsh -55/125 °C temperature cycle used in this study results in a large global shear strain per cycle of 2.6% for the 192CABGA and 1.2% for the 84CTBGA, as demonstrated by SI-Fig. 4 in Supplementary Information. Consistent with these factors, the most extensive continuous cracking along the substrate/IMC layer interface was measured in the highest-Bi solder, Sn-2.3Ag-0.5Cu-6.0Bi, coupled with the highest strain package, 192CABGA (Fig. 9), which resulted in worse thermal cycling performance than Sn-3.0Ag-0.5Cu (Fig. 6(b)).

**Fig. 11:**
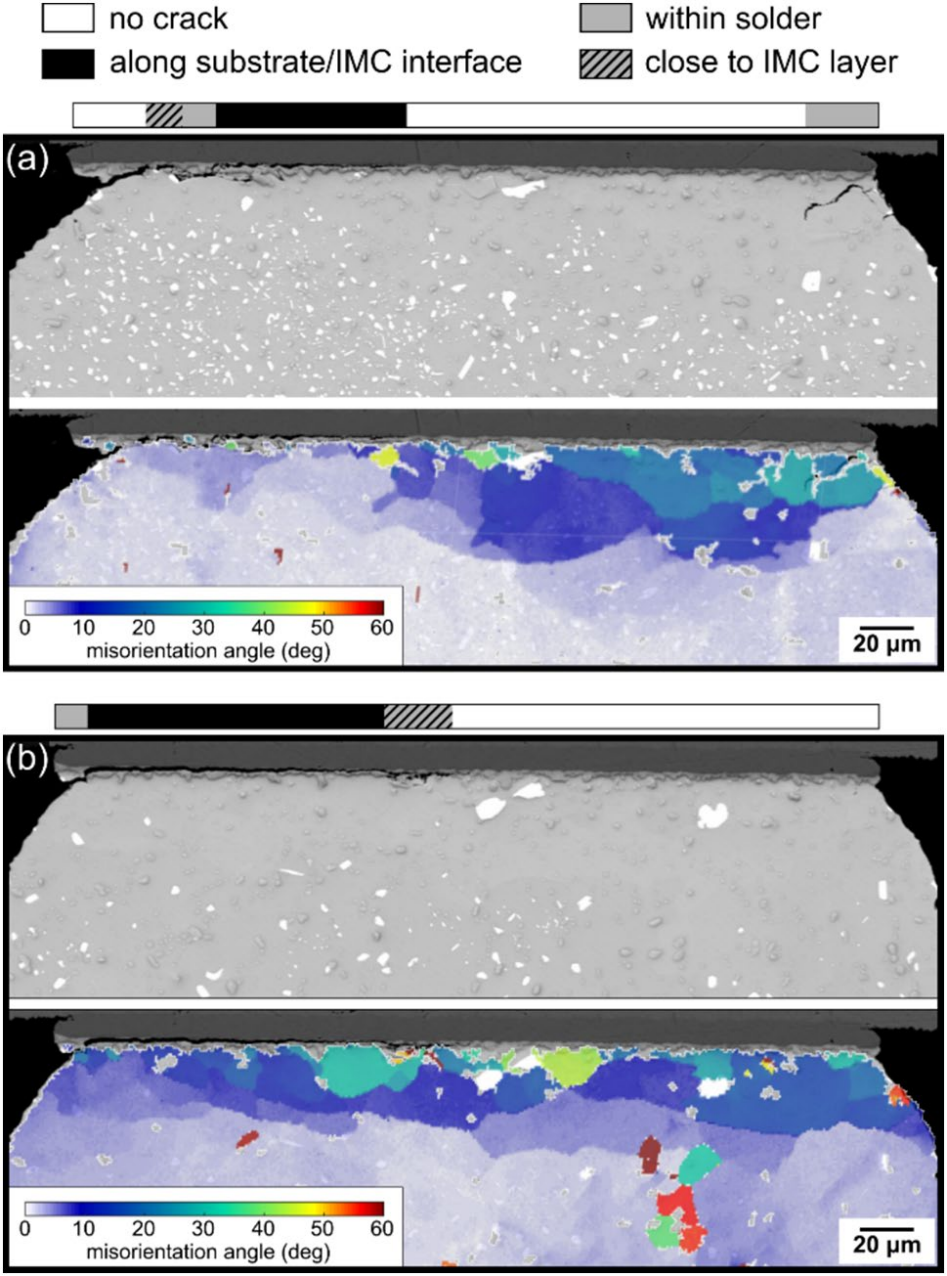
The two Sn-2.3Ag-0.5Cu-6.0Bi 84CTBGA joints, **a** M3 and **b** M5, with some cracking along the substrate/IMC interface (see Fig. 9). EBSD β-Sn misorientation maps show extensive areas of MO and recrystallisation in these cases

Returning to Fig. 9, note that two Sn-2.3Ag-0.5Cu-6.0Bi joints in the 84CTBGA—M3 and M5—had continuous crack paths along the substrate/IMC layer interface. Since both the joints are not fully cracked, it is natural to ask why continuous cracking along the substrate/IMC layer interface in the 84CTBGA of Sn-2.3Ag-0.5Cu-6.0Bi did not lead to early failure whereas those in the 192CABGA package did. To explore this, Fig. 11 shows the SEM images and EBSD misorientation maps of the joints M3 (Fig. 11(a)) and M5 (Fig. 11(b)) in the 84CTBGA. In both joints, we see substantial misorientation in the β-Sn plus some recrystallisation, indicating significant β-Sn plasticity and energy dissipation in the solder. In contrast, in the fully cracked 192CABGA joint with long and continuous cracking along the substrate/IMC layer interface (Fig. 10(b)), there is little misorientation and no recrystallisation of β-Sn. Thus, while these two joints in the 84CTBGA had continuous crack paths along the substrate/IMC layer interface, they also dissipated energy plastically in the solder which will have reduced the stress transfer to the IMC layer.

In summary, the fatigue behaviour of the solder joints depended on the combination of Bi content and the electronic package type. Adding 3 to 6 wt.% bismuth to near-eutectic Sn–Ag–Cu solders increases solder strength and, in most cases, enhanced the thermal fatigue reliability. However, the highest-Bi solder (Sn-2.3Ag-0.5Cu-6.0Bi) has the highest yield/flow stress, which limits the deformation of the solder. This, coupled with the higher strain package (192CABGA) and harsher temperature cycling (-55/125 °C), resulted in stress transfer to the IMC layer, extensive cracking along the substrate/IMC layer interface, and a reduced solder joint life.

### (Bi) precipitates in thermally cycled Sn-2.3Ag-0.5Cu-6.0Bi joints

During -55/125 °C thermal cycling, the Sn–Ag–Cu-Bi solder joints are cyclically heated above and then cooled below the solvus of Bi in β-Sn (Fig. 5(c)). Thus, if full equilibrium was reached, (Bi) phase would fully dissolve in the heating cycle and reprecipitate in the cooling cycle. In this study, no (Bi) phase was detected in the Sn-3.0Ag-0.8Cu-3.0Bi and Sn-4.0Ag-0.6Cu-3.5Bi joints after thermal cycling. In contrast, (Bi) precipitates were observed in Sn-2.3Ag-0.5Cu-6.0Bi joints after thermal cycling. Two orientation relationships (ORs) of the (Bi) precipitates were measured with EBSD which are the same as those reported by Belyakov et al. [71] in Sn-3.0Ag-0.5Cu-4.0Bi:

**Table 1:** Summary of SEM–EDX point measurements within the matrix in two solder alloys after thermal cycling. In Sn-2.3Ag-0.5Cu-6.0Bi, the recrystallised grains correspond to the PFZs. S.D. = standard deviation. N = number of EDX measurement points

	Recrystallized β-Sn grains			Bulk β-Sn grains		
	mean	S.D	N	mean	S.D	N
	[wt.% Bi]	[wt.% Bi]	[-]	[wt.% Bi]	[wt.% Bi]	[-]
Sn-4.0Ag-0.6Cu-3.5Bi	3.7	0.2	17	3.8	0.2	16
Sn-2.3Ag-0.5Cu-6.0Bi	3.9	0.3	32	5.0	0.4	31

OR-1 $$\left\{ {1 0 0} \right\}_{\beta Sn} \left\| {\left\{ {0 1 \overline{1} 2} \right\}_{{\left( {Bi} \right)}} } \right.\,and \,\left\langle {0 0 1} \right\rangle_{\beta Sn} \left\| {\left\langle {2 \overline{2} 0 1} \right\rangle } \right._{{\left( {Bi} \right)}}$$

OR-2 $$\left\{ {1 0 0} \right\}_{\beta Sn} \left\| {\left\{ {0 1 \overline{1} 2} \right\}_{{\left( {Bi} \right)}} } \right.\,and \,\left\langle {0 1 1} \right\rangle_{\beta Sn} \left\| {\left\langle {2 \overline{2} 0 1} \right\rangle } \right._{{\left( {Bi} \right)}}$$

**Fig. 12:**
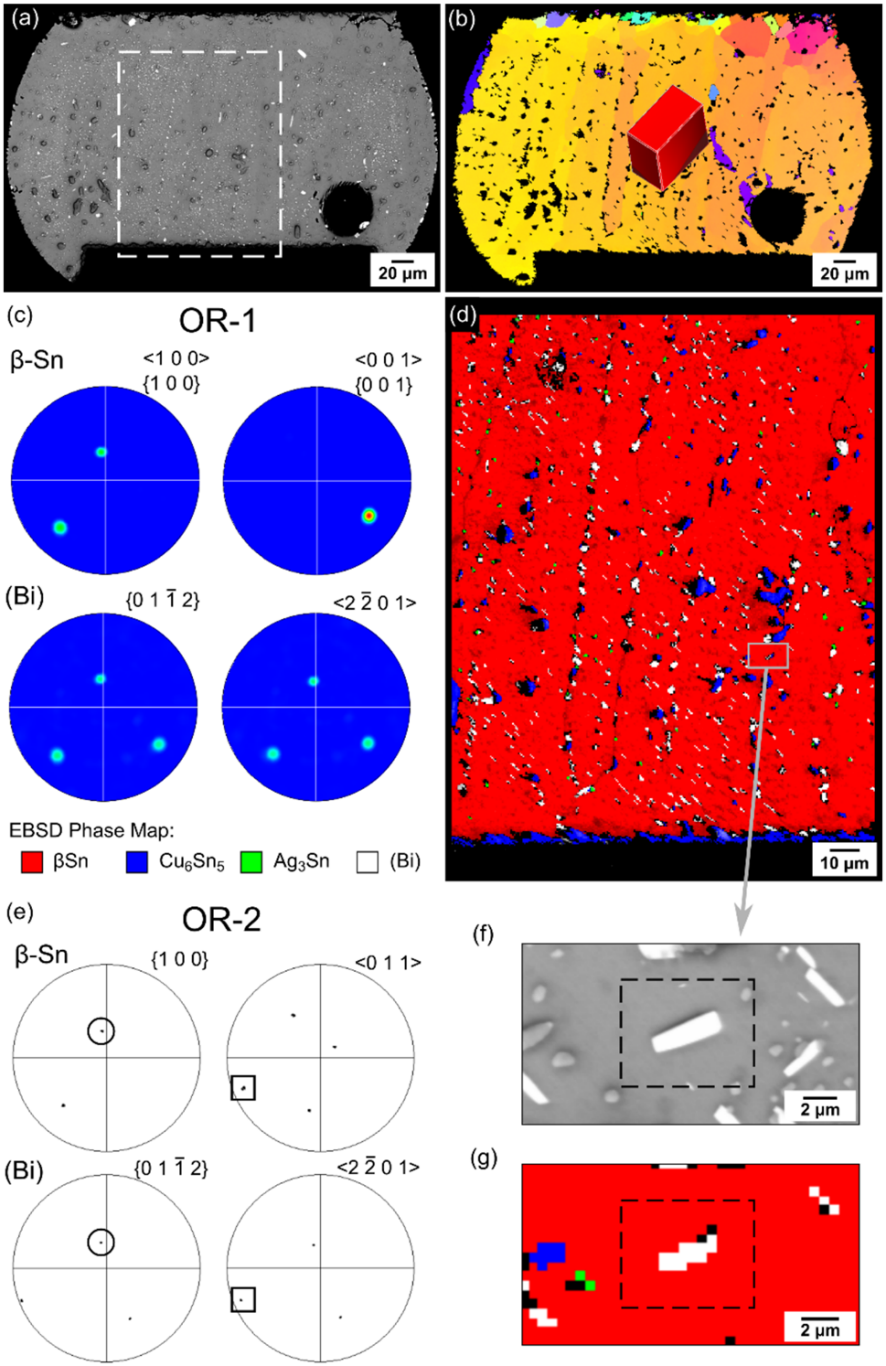
Orientation relationships (ORs) between the β-Sn matrix and (Bi) precipitates in a thermally cycled Sn-2.3Ag-0.5Cu-6.0Bi joint. **a** Backscatter SEM image and **b** EBSD IPF-Y map of the joint. **c** Pole figures showing OR-1 between (Bi) and β-Sn across the boxed area in **a**. **d** EBSD phase map of the boxed area in **a**. **e** Pole figures from a (Bi) plate with OR-2. **f** Backscatter SEM image and **g** EBSD phase map of the (Bi) plate with OR-2 (in the dashed box)

As demonstrated in Fig. 12, the majority of (Bi) plates had OR-1 with β-Sn in thermally cycled Sn-2.3Ag-0.5Cu-6.0Bi joints. Fig. 12(d) shows an EBSD phase map of a large region containing more than 500 (Bi) particles. The pole figures in Fig. 12(c) summarise orientations in this whole area in a convolution plot. There is a single β-Sn grain, and the majority of (Bi) particles have OR-1 with the β-Sn grain. Note, however, that a convolution plot hides minority orientations and not all (Bi) particles have OR-1. Searching individual (Bi) plates also revealed plates with OR-2 such as the example given in Fig. 12(e), (f), and (g).

**Fig. 13:**
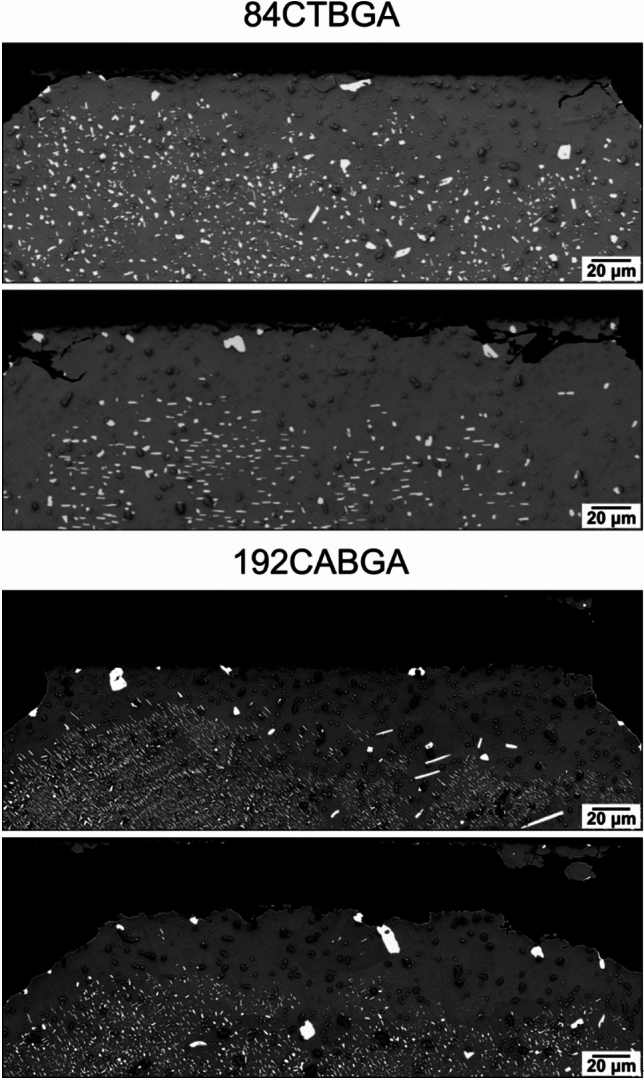
(Bi) precipitate-free zones (PFZs) in the top part of Sn-2.3Ag-0.5Cu-6.0Bi joints in the 84CTBGA (top) and 192CABGA (bottom) packages after thermal cycling

After 4876 thermal cycles, most Sn-2.3Ag-0.5Cu-6.0Bi joints contained (Bi) precipitate-free zones (PFZs) in the β-Sn near the top substrate where strain localises. Fig. 13 shows four joints with PFZs as examples, where fine (Bi) precipitates were distributed across the bulk areas of the joints and, near the top substrate, there were regions of β-Sn with no (Bi) precipitates and a few large (Bi) particles in between.

**Fig. 14:**
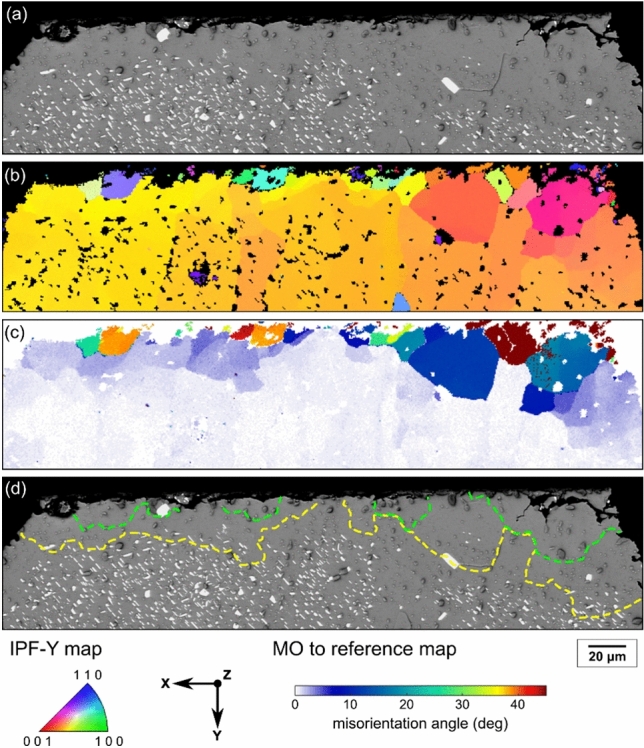
PFZ microstructures at the top part of a Sn-2.3Ag-0.5Cu-6.0Bi joint. **a** Backscattered SEM image, **b** EBSD IPF-Y map, and **c** β-Sn misorientation to parent grain map of the same area. **d** β-Sn grain boundaries from EBSD overlaid on the SEM image. Yellow boundaries have misorientations 5°-15°; green boundaries > 15°

To provide insights into the location of (Bi) precipitate-free zones, Fig. 14 presents EBSD orientation mapping and the BSE image of a typical Sn-2.3Ag-0.5Cu-6.0Bi joint in a 84CTBGA package. The solder joint can be divided into three regions based on the misorientation to the parent β-Sn grain: (i) a low deformation area, with misorientation < 5°, below the yellow dashed line in Fig. 14(d) corresponding to white misorientations in Fig. 14(c); (ii) a region of subgrains with misorientation between 5° and 15° between the green and yellow dashed lines in Fig. 14(d) corresponding to light-blue and dark-blue misorientations in Fig. 14(c); and (iii) a region of recrystallization with misorientation > 15° above the green dashed line in Fig. 14(d) corresponding to hotter colour misorientations in Fig. 14(c). Examining Fig. 14, we see that the (Bi) precipitate-free zones generally correspond to recrystallized grains and some subgrains (above the yellow dashed lines in Fig. 14(d)), and the large (Bi) particles between the precipitate-free zones are generally at recrystallised grain boundaries or subgrain boundaries.

**Fig. 15:**
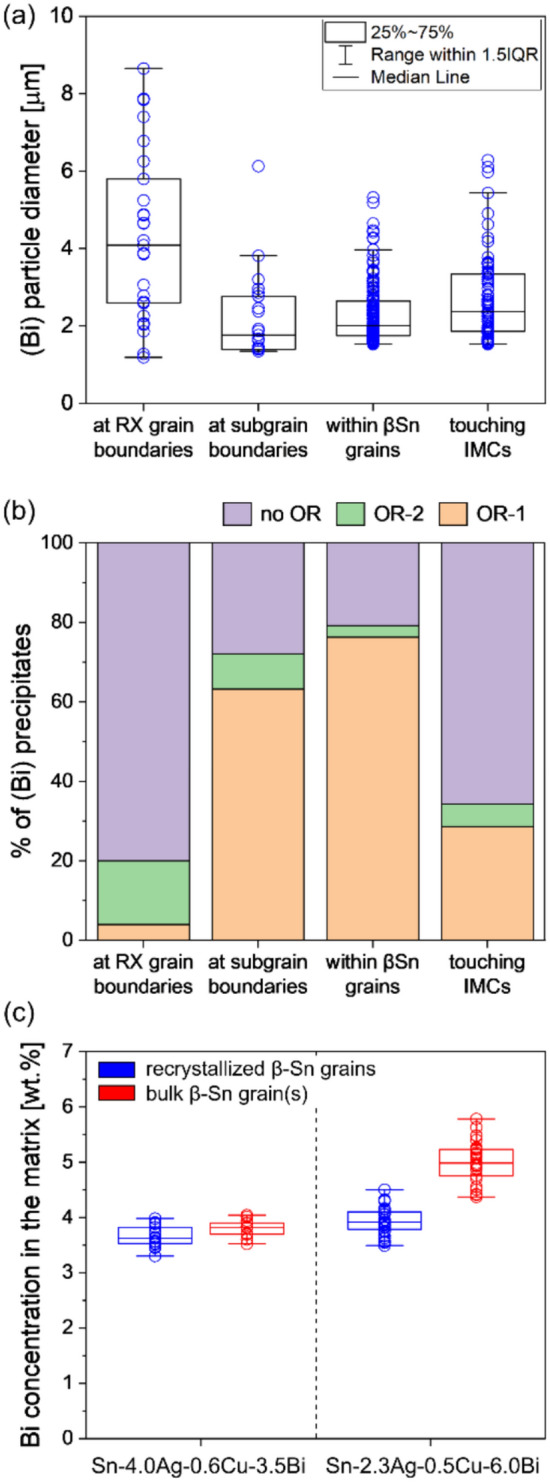
**a** Box plot of (Bi) particle diameters in different locations in Sn-2.3Ag-0.5Cu-6.0Bi. **b** Stacked column chart of the % of (Bi) particles with OR-1 and OR-2 in different locations. **c** Box plot of SEM–EDX point-measured Bi concentration in recrystallized β-Sn grains and bulk β-Sn grain(s) in two solder alloys. N.B. the matrix contains some (Bi) precipitates in the bulk grains of Sn-2.3Ag-0.5Cu-6.0Bi joints. All data are for 84CTBGA packages after thermal cycling

To further quantify the location of the large (Bi) particles, Fig. 15(a) shows the dependence of (Bi) precipitate size on the location in the microstructure. 212 (Bi) precipitates in five Sn-2.3Ag-0.5Cu-6.0Bi solder joints were analysed by combining EBSD maps and BSE images of the same areas. Data were separated into four categories: (i) (Bi) at β-Sn recrystallised (RX) grain boundaries (misorientation (MO) > 15°); (ii) (Bi) at subgrain boundaries (5° < MO < 15°); (iii) (Bi) within β-Sn grains; and (iv) (Bi) sharing an interface with Cu_6_Sn_5_ or Ag_3_Sn. The median and maximum diameters of (Bi) particles “at RX boundaries” are significantly larger than those of the other three groups, indicating that recrystallised grain boundaries promoted the coarsening of (Bi) precipitates during thermal cycling. The tendency of large (Bi) particles to be at recrystallised grain boundaries is likely because high angle grain boundaries provide faster diffusion paths for Bi atoms, accelerating the coarsening of (Bi) particles on recrystallised grain boundaries.

Fig. 15(b) shows the dependence of the orientation relationship between (Bi) precipitates and the surrounding β-Sn on the location in the microstructure. Within β-Sn grains, ~ 80% of (Bi) particles had OR-1 or OR-2 with β-Sn and there were many more (Bi) particles with OR-1 than those with OR-2. This is consistent with reference [71] where OR-1 was shown to have better interfacial coherency than OR-2. ~ 70% of (Bi) particles located at subgrain boundaries had OR-1 or OR-2 with β-Sn (Fig. 15(b)), indicating that the OR was maintained at subgrain boundaries in most cases. In contrast, (Bi) particles located at recrystallised grain boundaries only had OR-1 or OR-2 with β-Sn for ~ 20% of particles. This was probably because, while a (Bi) particle may have had OR-1 or OR-2 with a β-Sn grain when it just precipitated, the (Bi) particle lost its OR when the β-Sn grain continued to recrystallize, resulting in incoherent (Bi) particles at recrystallised grain boundaries.

Fig. 15(b) also shows that less than 40% of (Bi) precipitates had an OR with β-Sn when they touched an IMC particle (Ag_3_Sn or Cu_6_Sn_5_). These (Bi) precipitates without an OR with β-Sn may have nucleated on the IMC particles rather than within the β-Sn but this was not studied in detail here.

To investigate the effect of large (Bi) particles at recrystallised grain boundaries on the Bi concentration in the recrystallized β-Sn grains (i.e. in the PFZs), EDX point measurements were taken in the recrystallized β-Sn grains and in the bulk β-Sn grain(s) of thermally cycled Sn-4.0Ag-0.6Cu-3.5Bi and Sn-2.3Ag-0.5Cu-6.0Bi joints in the 84CTBGA. The results are presented in Fig. 15(c) and Table 1.

In Sn-4.0Ag-0.6Cu-3.5Bi, no (Bi) phase was detected, and both the recrystallized β-Sn grains and the bulk β-Sn grain(s) had Bi concentration near to 3.75 wt.%, corresponding to the calculated Bi concentration in β-Sn for Sn-4.0Ag-0.6Cu-3.5Bi at 23 °C (accounting for the near-zero solubility of Bi in Ag_3_Sn and Cu_6_Sn_5_) [72]. In Sn-2.3Ag-0.5Cu-6.0Bi, the Bi concentration in the recrystallized β-Sn grains is only slightly (~ 0.2 wt.%) higher than in Sn-4.0Ag-0.6Cu-3.5Bi. This shows that the accumulation of Bi atoms into large (Bi) particles at RX boundaries in Sn-2.3Ag-0.5Cu-6.0Bi leaves PFZs that are depleted in Bi. Also, in Sn-2.3Ag-0.5Cu-6.0Bi, the Bi concentration is significantly higher in the bulk grain(s) than in the recrystallized β-Sn grains (Fig. 15(c)). This is likely due to the small (Bi) precipitates widely distributed in the bulk grain(s) of Sn-2.3Ag-0.5Cu-6.0Bi, which, when contained in the electron interaction volume during EDX measurements, increased the measured Bi concentration beyond the equilibrium concentration of Bi in β-Sn at 23 °C. The mean value of this region, however, is still lower than 6 wt.% (Table 1) since there were already some large (Bi) particles before polishing, and the point measurements intentionally avoided these large (Bi) particles.

The precipitation and accumulation of (Bi) particles at RX boundaries and the development of (Bi) precipitate-free zones in recrystallised grains and subgrains are likely to have complex effects on the failure mechanisms in thermal fatigue. For example, precipitate-free grains will be softer than β-Sn regions containing fine (Bi) precipitates which would be expected to further promote localised deformation within recrystallised grains and subgrains. At the same time, grain boundary (Bi) precipitates may pin recrystallised grain boundaries and reduce grain boundary sliding (e.g. [73, 74]) which is an important part of the failure mechanism in the thermal fatigue of Sn–Ag–Cu-based solders [21, 53, 54, 75, 76]. Such a pinning effect depends on the size of grain boundary (Bi) precipitates which change during thermal cycling and subsequent storage. Further work is required to examine this complexity.

It is interesting to note in Fig. 1 and Fig. 6, that the SAC + Bi solders containing no (Bi) precipitates after -55/125 °C thermal cycling (Sn-3.0Ag-0.8Cu-3.0Bi and Sn-4.0Ag-0.6Cu-3.5Bi) performed better than the solder containing (Bi) precipitates (Sn-2.3Ag-0.5Cu-6.0Bi). While there are multiple aspects to this performance difference, it can be concluded that (Bi) precipitates are not required to achieve high reliability in a SAC + Bi solder.

## Conclusions

Microstructure evolution, thermal cycling performance and failure mechanisms have been investigated in high reliability Sn–Ag–Cu-Bi solder joints. Three Sn–Ag–Cu-Bi solders (Sn-3.0Ag-0.8Cu-3.0Bi, Sn-4.0Ag-0.6Cu-3.5Bi, and Sn-2.3Ag-0.5Cu-6.0Bi) and the widely used Sn-3.0Ag-0.5Cu (SAC305) solder have been compared in harsh thermal cycling from -55/125 °C in two BGA package types: an 84CTBGA and a 192CABGA. The following new insights have been identified.

1. After soldering, the Bi additions promoted the formation of single grain solder joints and suppressed interlaced microstructures in multi-grain joints.
2. In all as-soldered SAC + Bi joints, some (Bi) phase was present due to a nonequilibrium (Scheil) eutectic reaction and there were steep Bi concentration gradients in the β-Sn phase. During four years of storage at room temperature, the volume fraction of the (Bi) phase reduced significantly due to partial solutionising and homogenisation.
3. The relative performance of the Sn–Ag–Cu-Bi solders compared with Sn-3.0Ag-0.5Cu depended on the solder composition and the electronic package type used for the ATC test vehicle. In the lower strain 84CTBGA package, all three Bi-containing solders significantly outperformed Sn-3.0Ag-0.5Cu. In the 192CABGA package, the Sn-3.0Ag-0.8Cu-3.0Bi and Sn-4.0Ag-0.6Cu-3.5Bi solders outperformed Sn-3.0Ag-0.5Cu but by a smaller margin than in the 84CTBGA. Whereas the Sn-2.3Ag-0.5Cu-6.0Bi solder, with the highest Bi concentration, had lower characteristic life than Sn-3.0Ag-0.5Cu in the higher strain 192CABGA package. These effects could be correlated with EBSD measurements of the accumulated deformation of β-Sn in the solder and the resulting crack path.
4. Whereas Sn-3.0Ag-0.5Cu generally failed by solder fatigue involving crack paths in the β-Sn, the Sn–Ag–Cu-Bi solders commonly had more complicated crack paths involving mixtures of (i) crack paths in the β-Sn, (ii) crack paths in the β-Sn very close to the IMC layer, and/or (iii) crack paths along the substrate/IMC layer interface. The Sn–Ag–Cu-Bi solders that performed best had mixed-mode cracks that either (i) alternated repeatedly between the solder and IMC layer, or (ii) had long crack paths within the solder, both of which involved significant plastic strain and recrystallisation in the β-Sn. The worst performing combination (the Sn-2.3Ag-0.5Cu-6.0Bi solder in the 192CABGA package) had long, continuous cracks along the substrate/IMC layer interface, limited misorientation in β-Sn and no recrystallization. Thus, high strength Sn–Ag–Cu-Bi solders can improve the resistance to thermal fatigue in BGA packages, but high solder strength needs to be balanced against excessive stress transfer to the IMC layer.
5. Sn-2.3Ag-0.5Cu-6.0Bi joints contained (Bi) precipitates after thermal cycling which interacted with the failure mechanism. In the region of strain localisation, recrystallised grains and subgrains were (Bi) precipitate-free zones with relatively low Bi concentration, and anomalously large (Bi) precipitates were located at recrystallised grain boundaries. This adds extra complexity to the failure mechanism in ‘high Bi’ Sn–Ag–Cu-Bi solders, with an additional softening mechanism in recrystallised grains and the possibility of (Bi) particles pinning recrystallised grain boundaries.
6. The findings in this works provide guidelines for developing high reliability Sn–Ag–Cu-Bi solders. To optimize the reliability of SAC + Bi solders under harsh thermal cycling, Bi content must be carefully controlled to balance solder strength and plasticity. If the Bi content is too low (< 2 wt.%), the solder lacks sufficient strength to resist thermal fatigue, resulting in early joint failure. Conversely, if the Bi content is too high (> 5 wt.%), the solder exhibits limited plastic deformation, causing extensive and continuous cracking at the IMC layer interface and, consequently, premature joint failure. Overall, Sn–Ag–Cu-Bi solders with 3 to 4 wt.% Bi exhibited the optimum thermal cycling reliability, and are most suitable for applications under harsh environments.

## Supplementary Information

Below is the link to the electronic supplementary material.

Supplementary file1 (DOCX 5451 KB)

## Data Availability

Datasets including EBSD maps and SEM images will be made available on request.
