# Supplementary material for: Effects of bismuth on the microstructure and crack propagation in Sn–Ag–Cu-Bi solder joints during thermal cycling
Source: J Mater Sci Mater Electron. 2025 Oct 24;36(30):1926. doi: 10.1007/s10854-025-15979-2 (PMC12552320; doi:10.1007/s10854-025-15979-2)
Supplement: Supplementary file 1 — Supplementary file1 (DOCX 5451 KB) [file 10854_2025_15979_MOESM1_ESM.docx]

**Supplementary Information**

Effects of bismuth on the microstructure and crack propagation in Sn-Ag-Cu-Bi solder joints during thermal cycling

C.L. Hsieh^1*^, R.J. Coyle^2^, S.A. Belyakov^1^, J.W. Xian^1^, C.M. Gourlay^1**^

^1^ Department of Materials, Imperial College London, London. SW7 2AZ. UK

^2^ Nokia Bell Labs, Murray Hill, NJ, USA

**Details of the thermal cycling test vehicle**

| 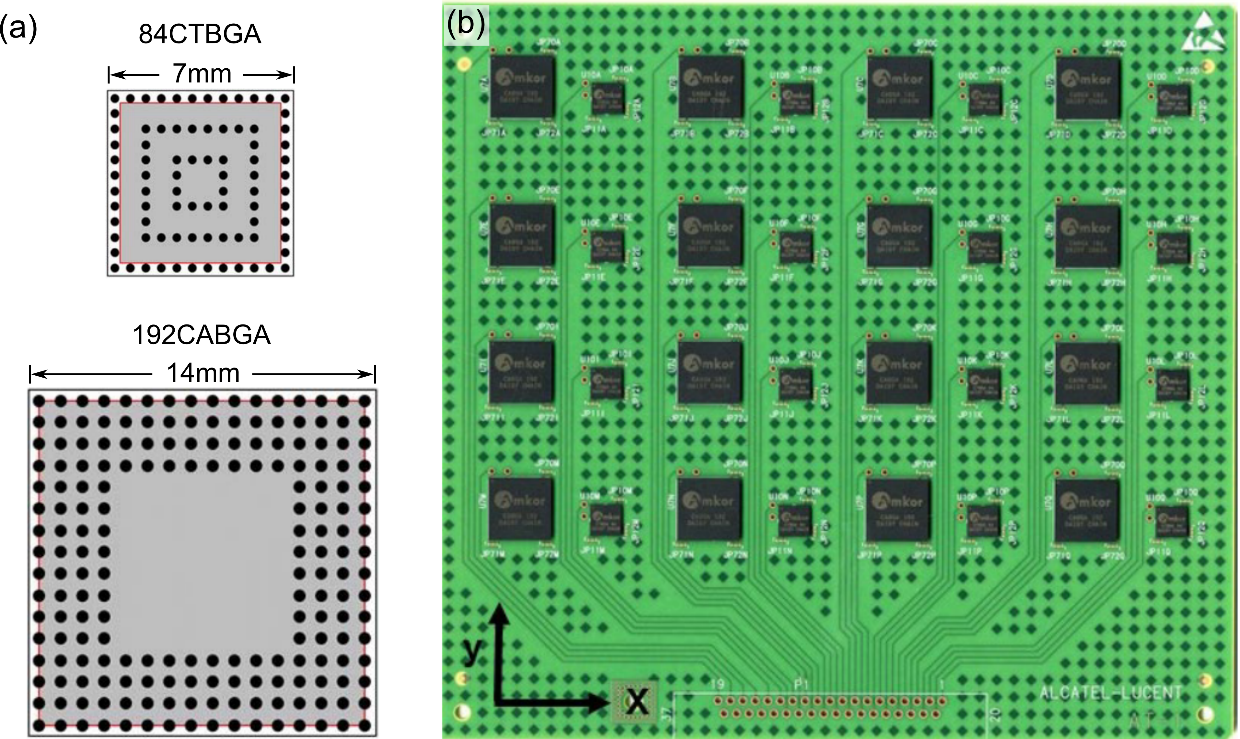  SI-Figure 1 (a) Pin diagrams of 84CTBGA and 192CABGA packages. (b) The thermal cycling test vehicle used in this study, where 16 of 84CTBGA packages and 16 of 192CABGA packages were populated on a PCB. Reproduced from Ref. [1]. |
| --- |

SI-Table 1 Attributes of BGA packages and PCB used in this study. Reproduced from Ref. [1].

| **BGA package attributes** | | |
| --- | --- | --- |
| **Designation** | 192CABGA | 84CTBGA |
| **Die size** | 12 x 12 mm | 5 x 5 mm |
| **Package size** | 14 x 14 mm | 7 x 7 mm |
| **Ball array** | 16 x 16 | 12 x 12 |
| **Ball pitch** | 0.8 mm | 0.5 mm |
| **Ball diameter** | 0.46 mm | 0.3 mm |
| **Solder joint height** | 0.31 mm | 0.2 mm |
| **Pad diameter** | 0.381 mm | 0.3 mm |
| **Pad finish** | Electrolytic Ni/Au | Electrolytic Ni/Au |
| **Distance from neutral point to corner joint (DNP_max_)** | 8.49 mm | 3.89 mm |
| **Mean CTE** | 9.4 ppm/K | 11.0 ppm/K |
| **PCB attributes** | | |
| **Dimensions** | 165 x 178 x 2.36 mm | |
| **Laminate** | Panasonic R-1755V | |
| **Surface finish** | Entek HT OSP | |
| **No. of Cu layers** | 6 | |
| **Pad diameter** | 0.356 mm | 0.254 mm |
| **Solder mask diameter** | 0.483 mm | 0.381 mm |

**Microstructure of Sn-4.0Ag-0.6Cu-3.5Bi joints at room temperature**

| 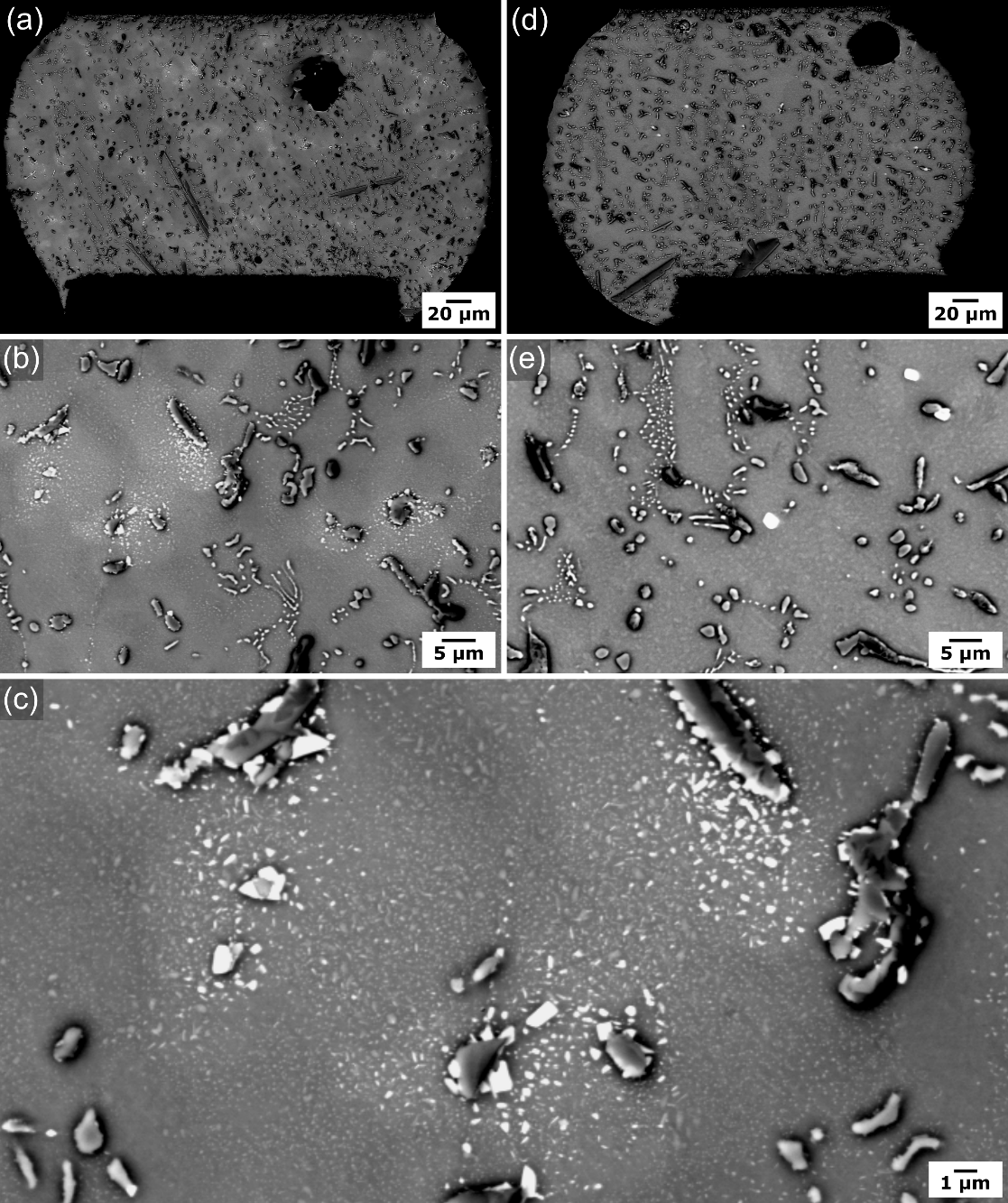  SI-Figure 2 Microstructure evolution at room temperature in Sn-4.0Ag-0.6Cu-3.5Bi joints. (a)-(c) 20 hours after reflow soldering. (d)-(e) after 4 years storage at room temperature. |
| --- |

**Microstructure of Sn-2.3Ag-0.5Cu-6.0Bi joints at room temperature**

| 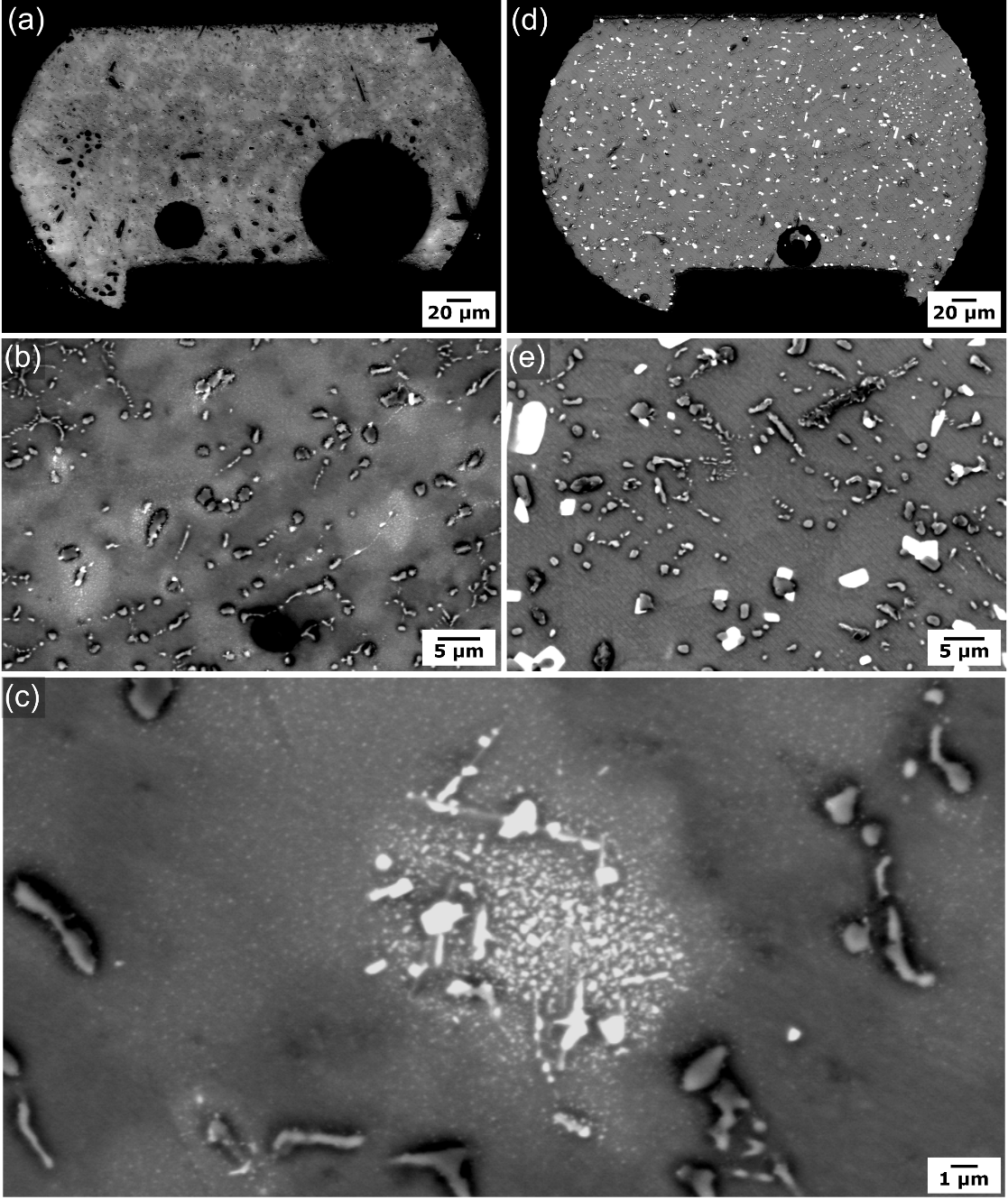  SI-Figure 3 Microstructure evolution at room temperature in Sn-2.3Ag-0.5Cu-6.0Bi joints. (a)-(c) 20 hours after reflow soldering. (d)-(e) after 4 years storage at room temperature. |
| --- |

**Maximum shear strain per thermal cycle**

| 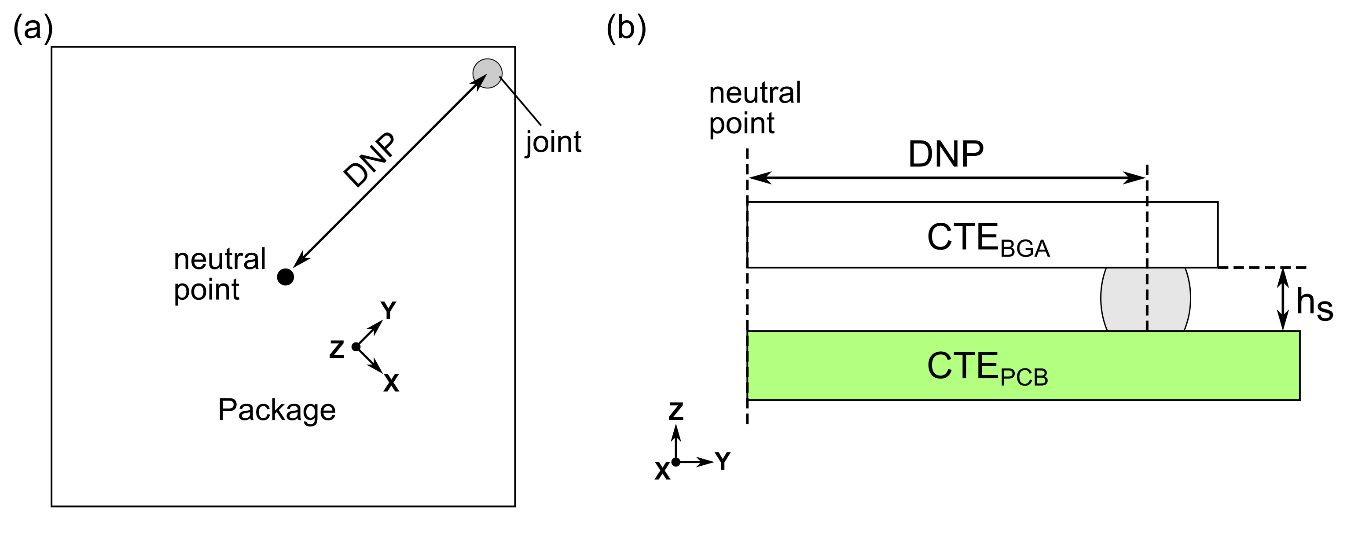  SI-Figure 4 (a) Top view and (b) cross-section of the test vehicle, illustrating the key parameters to the shear strain of solder joints due to the CTE mismatch between the BGA package and the PCB during thermal cycling. According to (b), the maximum shear strain that a joint undergoes during thermal cycling can be expressed as [2]:  $\gamma_{max}=\frac{DNP\cdot\left\vert{CTE}_{BGA}-{CTE}_{PCB} \right\vert\cdot\Delta T}{h_{s}}$  where $DNP$ is the distance between the joint and the neutral point (center) of the package, ${CTE}_{BGA}$ and ${CTE}_{PCB}$ are the thermal expansion coefficients of the BGA package and the PCB respectively, $h_{s}$ is the height of the joint, and $\Delta T$ is the temperature range between the cold and hot sides of a thermal cycle, which is 180°C for the -55/125°C thermal cycling test in this study. Based on this equation, the largest shear strain occurs on the joints at the corners of a package, which has the largest $DNP$. By taking the values of ${DNP}_{max}$, ${CTE}_{BGA}$, ${CTE}_{PCB}$, $h_{s}$ from SI-Table 1 into the above equation, the maximum shear strains per thermal cycle for solder joints in 84CTBGA and 192CABGA packages are 1.348% and 2.643% respectively. |
| --- |

**Reference**

[1] R. Coyle *et al.*, “Thermal cycling reliability and failure mode of two ball grid array packages with high reliability Pb-free solder alloys,” in *SMTA International*, Rosemont, IL, USA, 2019, pp. 439–456.

[2] J.-P. M. Clech, R. J. Coyle, and B. Arfaei, “Pb-Free Solder Joint Thermo-Mechanical Modeling: State of the Art and Challenges,” *JOM*, vol. 71, no. 1, pp. 143–157, Jan. 2019, doi: 10.1007/s11837-018-3003-0.
